# Sex hormone-binding globulin improves lipid metabolism and reduces inflammation in subcutaneous adipose tissue of metabolic syndrome-affected horses

**DOI:** 10.3389/fmolb.2023.1214961

**Published:** 2023-12-11

**Authors:** Lynda Bourebaba, Martyna Kępska, Badr Qasem, Magdalena Zyzak, Jacek Łyczko, Marta Klemens, Malwina Mularczyk, Krzysztof Marycz

**Affiliations:** ^1^ Department of Experimental Biology, Faculty of Biology and Animal Science, Wrocław University of Environmental and Life Sciences, Wrocław, Poland; ^2^ Department of Food Chemistry and Biocatalysis, Faculty of Biology and Animal Science, Wrocław University of Environmental and Life Sciences, Wrocław, Poland; ^3^ International Institute of Translational Medicine, Wisznia Mała, Poland; ^4^ Department of Surgical and Radiological Sciences, School of Veterinary Medicine, University of California, Davis, Davis, CA, United States

**Keywords:** EMS, SHBG, lipid metabolism, metaflammation, lipidome, insulin signaling

## Abstract

Equine metabolic syndrome (EMS) is a steadily growing endocrine disorder representing a real challenge in veterinary practice. As a multifactorial condition, EMS is characterized by three main metabolic abnormalities including insulin resistance, increased adiposity or obesity and hoof laminitis. Adipose tissue dysfunction is recognized as a core pathophysiological determinant of EMS, as it strongly participates to lipotoxicity and systemic metaflammation, both of which have been closely linked to the development of generalized insulin resistance. Besides, sex hormone binding globulin (SHBG) is an important sex steroids transporters that has been recently proposed as an important metabolic mediator. Therefore, the aim of this study was to verify whether SHBG treatment may ameliorate subcutaneous adipose tissue metabolic failure under EMS condition in terms of lipidome homeostasis, lipid metabolism programs, insulin signalling and local inflammation. Subcutaneous adipose tissue (SAT) biopsies were collected post-mortem from healthy (*n* = 3) and EMS (*n* = 3) slaughtered horses. SHBG protein has been applied to SAT samples from EMS horses for 24 h at a final concentration of 50 nM, while control groups (healthy and untreated EMS) were cultured in the presence of SHBG-vehicle only. Tissues from all groups were afterwards secured for downstream analysis of gene expression using RT-qPCR, protein levels by Western blot and ELISA assay and lipidomics through GC-MS technique. Obtained results showcased that SHBG intervention efficiently normalized the altered fatty acids (FAs) profiles by lowering the accumulation of saturated and trans FAs, as well as the pro-inflammatory arachidonic and linoleic acids. Moreover, SHBG showed promising value for the regulation of adipocyte lipolysis and engorgement by lowering the levels of perilipin-1. SHBG exerted moderated effect toward SCD1 and FASN enzymes expression, but increased the LPL abundance. Interestingly, SHBG exhibited a negative regulatory effect on pro-adipogenic stimulators and induced higher expression of KLF3, IRF3 and β-catenin, known as strong adipogenesis repressors. Finally, SHBG protein showed remarkable ability in restoring the insulin signal transduction, IR/IRS/Pi3K/AKT phosphorylation events and GLUT4 transporter abundance, and further attenuate pro-inflammatory response by lowering IL-6 tissue levels and targeting the PDIA3/ERK axis. Overall, the obtained data clearly demonstrate the benefice of SHBG treatment in the regulation of adipose tissue metabolism in the course of EMS and provide new insights for the development of molecular therapies with potential translational application to human metabolic disorders.

## 1 Introduction

Metabolic syndrome (MetS) or syndrome X is defined as a cluster of metabolic defects comprising insulin resistance, hypertension, dyslipidaemia, central obesity or regional adiposity. The pathogenesis of MetS englobes both genetic and environmental factors; however, lifestyle seems to be an equally important risk factor, especially when insulin resistance related to obesity and inflammation are considered (Rochlani et al., 2017). MetS is nowadays becoming a frequently diagnosed endocrine disorder not only in human beings but also among animals including horses. The pathogenesis of equine metabolic syndrome (EMS) reflects many similarities to MetS and therefore becomes a valuable large animal model for studying endocrine disorders. Indeed, EMS condition has been recognized as a similar endocrinopathy to human Metabolic Syndrome (MetS). Statements from the American College of Veterinary Internal Medicine (ACVIM) and the European College of Equine Internal Medicine (ECEIM) EMS consensus have highlighted the clinical, phenotypic, and molecular resemblances between EMS and the pathophysiology of human MetS. Shared mechanisms of these conditions involve irregular insulin regulation and resistance, abnormal fat distribution, and metaflammation, offering promising opportunities for utilizing EMS as a valuable experimental model in translational human medicine (Frank et al., 2010; Morgan et al., 2015; Durham et al., 2019; Ragno et al., 2019).

In both MetS and EMS central obesity or adiposity is regarded as a major determinant of the disease development and a serious factor promoting local and systemic inflammation that is often associated with cardiovascular diseases development or laminitis in horses. The latter is recognized among insulin resistance horses as the most debilitating, life-threatening disorder that is often diagnosed among obese horses (Ertelt et al., 2014). According to the Center of Disease Control and Prevention (CDC), obesity prevalence has increased by 35% in the last 15 years in the United States (Fahed et al., 2022). These alarming data are not alien to European countries where obesity is growing at a similar pace. Surprisingly, the same scenario has been observed among large animals; and obesity-related insulin resistance has been identified in more than 30% of horses in the territory of the United Kingdom. These data clearly show that obesity or adiposity is our century’s devastating disorder that has a serious impact on health and in consequence, becomes a significant financial burden for many healthcare systems around the world (Rendle et al., 2018; Krzysztoszek et al., 2019).

Obesity is actually recognized as a worldwide epidemic and one of the most prevalent risk factors for insulin resistance, immune-mediated disorders, non-alcoholic fatty liver disease, cardiovascular affections (CVD), several types of cancer, and type 2 diabetes mellitus (T2D). As above mentioned, obesity is strongly associated with the development of many other disorders, and as a multifactorial chronic disease is recognized as a key player in the development of MetS or EMS (Pi-Sunyer, 2009). Adipose tissue is considered an is a metabolically dynamic endocrine organ that actively produces and secretes various humoral factors (adipokines), hormones, and cytokines that play a fundamental role in local and systemic insulin dysregulation, and insulin resistance development as well as inflammation. Adipose tissue insulin resistance is a key factor strongly impacting fat hypertrophy, which is believed to be closely associated to low-grade local and systemic inflammation (Zatterale et al., 2020). Impaired glucose uptake within adipocytes that is mediated by hampered glucose transporter 4 (GLUT-4) translocation, combined with the lower expression and phosphorylation of adipocytes’ insulin receptor (IR) and its substrates (IRSs) have been found to play an important role in adipose tissue hyperinsulinemia and insulin resistance development in both human and horses (Reynolds et al., 2019; Ahmed et al., 2021). Metabolically impaired adipose tissue further displays altered lipid metabolism homeostasis, which subsequently aggravates the existing insulin resistance and metaflammation. Lipids play a pivotal role in the pathogenesis of insulin resistance and adipose tissue inflexibility. Once enlarged due to excess caloric intake, dysfunctional adipose tissue produces and releases toxic reactive lipids such as ceramides and diacyl-glycerides (DAG) that impede the insulin-mediated kinases phosphorylation and triggers various cellular stresses related to MAPK/NFκB pro-inflammatory pathway activation, excessive ROS generation, endoplasmic reticulum (ER) and mitochondrial disruption and cell apoptosis (Sears and Perry, 2015). Moreover, defects in lipolysis results in adipocytes engorgement, adipose tissue failure and excessive adipokines release, which promotes systemic insulin dysregulation (Guilherme et al., 2008). In EMS horses, adipocytes hypertrophy has been associated with increased expression of leptin and the inflammatory cytokines tumor necrosis factor (TNF)-α, IL1β and monocyte chemoattractant protein (MCP)-1 and typical fasting hyperinsulinemia, evoking the close relationship between flawed adipose tissue dynamics and insulin resistance progression (Reynolds et al., 2019).

Imbalanced saturated fatty acids levels deriving from cell surface-expressed lipases including the hormone-sensitive lipase (HSL) depletion exerts direct stimulatory feedback to activate various pro-inflammatory cascades leading to adipose tissue infiltration by macrophages, higher expression of TNF-α, MCP-1, and IL-6. Although the exact molecular mechanisms underlying metaflammation unleashing are quite complex, a body of evidence indicate that FFAs such as palmitic acid initiate a TLR4-dependent gene expression program that facilitate the NF-κB/MAPK inflammatory cascade (Kawai et al., 2021). Elevated expression of TNF-α, MCP-1, or IL-6 has been further reported to significantly reduce the expression of GLUT4 and promote the compensatory hyperinsulinemia. Moreover, pro-inflammatory cytokines are critically involved in the induction of energy expenditure by binding to signaling receptors in adipose tissue and activating leptin expression. More specifically, it was found that TNF-α is involved in the regulation of leptin expression and activation in adipose tissue of MetS individuals, and thus promotes adipose tissue hypoxia that modulates adipose depots expansion (Caputo et al., 2017). Massive expansion and remodeling of adipose tissue significantly contributes to vascular dysfunction including arterial stiffening. Excessive deposition of AT around inner organs and at the periphery promotes the excessive infiltration of immune cells and secretion of various cytokines and vasoconstrictor mediators (Koenen et al., 2021). Oxidative stress and proinflammatory processes associated with secreted cytokines and adipokines englobing TNF-α, IL-6, angiotensinogen, aldosterone-stimulating factors, aldosterone, adiponectin, dipeptidylpeptidase 4 (DPP-4), leptin, resistin, and MCP-1 arousing from dysfunctional AT have been evidenced as key players participating in obesity-related vascular dysfunction (Fleenor et al., 2022). Moreover, suppressed insulin metabolic signaling in endothelial cells triggers reductions in nitric oxide (NO) production within the vasculature, which further initiates vascular stiffening (Aroor et al., 2018). Noteworthy, besides visceral and subcutaneous fat have been evoked as critical contributors of vascular stiffening in the course of obesity and metabolic disorders, perivascular fat tissue surrounding blood vessels, was similarly found to exert strong paracrine effects influencing the vasculature plasticity and remodling. Hence, hypertrophic perivascular adipocytes release a number of mediators such as IL-6, resistin and visfatin that enhance the deposition of collagen type 1, advanced glycation end products and calcium phosphate in the vascular wall (Para et al., 2021).

Recently, the correlation between serum sex hormone binding globulin (SHBG), inflammation, and insulin resistance has been demonstrated in obese and MetS human patients (Alinezhad and Jafari, 2019). It was found, that low serum SHBG correspond to increased body mass index (BMI), excessive liver fat, chronic obstructive pulmonary disease and diabetes incidence (Simó et al., 2015; Wang, 2021). SHBG is a 373-amino-acid glycoprotein produced in the liver and adipose tissue and excreted to the bloodstream where it binds sex steroids to regulate their bioavailability, and as reported recently, SHBG acts as an anti-inflammatory mediator suppressing inflammation and lipid accumulation in macrophages and adipocytes (Yamazaki et al., 2018). Albeit the exact mechanisms underlying SHBG interaction with cells and tissues in a context independent from sex hormones transport remains still under debate, latest research suggested the existence of an SHBG-specific membrane-bound megalin receptor (R_SHBG_) which facilitates the endocytosis of SHBG. Interaction of SHBG with megalin has been proposed to activate a G-protein-coupled receptor second messenger system, which initiates the synthesis and release of cyclic adenosine monophosphate (cAMP) and subsequent activation of protein kinase A (Nakhla et al., 1999; Wallace et al., 2013). Interestingly, foregoing studies revealed that SHBG affinity to its receptor was highly decreased upon its fixation to sex steroids, and that the stronger the binding of a steroid to SHBG, the greater its capacity to hinder the interaction between SHBG and its receptor, suggesting the interplay of distinct signalling pathways mediating SHBG biological effects (Rosner et al., 2010). SHBG is thus gaining more attention as it might be a potential therapeutic factor in the treatment of obesity and associated inflammation and insulin resistance. Little is known regarding the eventual biological effects of SHBG in disease, however mounting evidence highlight the promising role of SHBG in reducing both oxidative and ER stress, which might be recognized as a primary mechanism involved in the modulation of adipose tissue insulin sensitivity (Kornicka-Garbowska et al., 2021). Although there is limited data documenting the direct effect of SHBG on adipose tissue and its molecular implications, recent investigation demonstrated that overexpression of SHBG in a model of SHBG-C57BL/ksJ-db/db mouse confers high protection against high fat diet-induced excessive weight gain while preventing metabolic profile deregulation by normalizing the glucose, insulin, leptin, resistin, adiponectin, cholesterol, FFA and TG plasma levels. Interestingly, the same study established a direct link between SHBG and adipose tissue turnover showing the ability of SHBG to activate lipolysis by promoting protein kinase A (PKA), extracellular signal-regulated kinase 1/2 (ERK-1/2), hormone-sensitive lipase (HSL) and perilipin (PLIN) expression and phosphorylation (Saez-Lopez et al., 2020). Noteworthy, our previously published findings illustrated similar effects of SHBG in HepG2 cells, where exposure of hepatocytes overloaded with palmitic acid to exogenous human SHBG resulted in a sharp decreased expression of FASN, ACLY, and peroxisome proliferator-activated receptor gamma (PPARγ), indicating the potential of SHBG to regulate liver metabolism by the inhibition of lipogenesis (Kornicka-Garbowska et al., 2021). Hence, it can be speculated that SHBG may exert favourable action on lipid metabolism in both liver and AT via similar mechanisms. Nevertheless, the exact molecular impact of SHBG on adipose tissue metabolism, inflammation and insulin resistance remains elusive, and more advanced investigations are needed to clarify the possible use of the glycoprotein as a therapeutic agent for metabolic disorders management.

For that purpose, this study aimed at investigating the impact of SHBG treatment on adipose tissue biopsies in terms of lipid metabolism and insulin sensitivity, and to further explore its role as a potential anti-inflammatory mediator in EMS condition. We have found that SHBG normalized the lipidome profile of EMS-derived adipose tissue, improved the insulin-kinases phosphorylation cascade and decreased the expression of IL-6 and MCP-1 mediated by the modulation of the PDIA3/ERK axis; however, further clinical trials are necessary to confirm the applicability and effectiveness of SHBG in counteracting EMS pathological events.

## 2 Materials and methods

### 2.1 Tissue samples collection

Subcutaneous Adipose Tissue (SAT) biopsies were collected post-mortem from Polish cold-blooded healthy and EMS horses from a local slaughterhouse (Targowa, Rawicz, Poland). Animals that were euthanized for reasons unrelated to this study, were aged between 8 and 10 years, and qualified by experienced veterinarians on the basis of their clinical profiles and medical history following previously described procedure and established criterions that included body weight (BW), body condition score (BCS), cresty neck score (CNS) and fasting insulin levels (Henneke et al., 1983; Basinska et al., 2015; Marycz et al., 2016). The clinical assessment data of qualified healthy horses (*n* = 3) and EMS horses (*n* = 3) are summarized in Table 1.

**TABLE 1:** Healthy and EMS horses’ qualification criteria.

Groups	Age	BW (kg)	BCS (1–9)	CNS (1–5)	Fasting insulin (mU/mL)	CGIT:GLU in 45 min (mg/dL)
**Healthy Horses**	8	569	6	1	8	74/n
	8	601	6	2	12	69/n
	10	542	7	2	10	73/n
**Average ± SD**	8.66 ± 1.15	570.66 ± 29.53	6.33 ± 0.57	1.66 ± 0.57	10 ± 2	72 ± 2.64
**EMS Horses**	9	710	8	3	83	144/p
	10	726	9	4	82	141/p
	10	760	9	5	89	137/p
**Average ± SD**	9.66 ± 0.57	732 ± 25.53	8.66 ± 0.57	4 ± 1	84.66 ± 3.78	140.5 ± 3.51

BW, body weight; BCS, body condition score; CNS, crest neck score; CGIT, combined glucose-insulin test; SD, standard deviation; GLU, glucose; p, positive test result; n, negative test result.

### 2.2 Preparation of subcutaneous adipose tissue specimens

Freshly collected tissue fragments were transported in Dulbecco′s Phosphate Buffered Saline (DPBS, Biowest) supplemented with 1% penicillin-streptomycin solution (PS, Biowest) to the cell culture facility. Within 2 h of collection, tissues were cut manually to smaller pieces (approximately 50 mg each) and washed twice in DPBS with 1% PS. The biopsies were transferred into 12-well plates containing a growth culture medium consisting of Dulbecco’s Modified Eagle’s Medium with 1,000 mg/L glucose, L-glutamine, and sodium bicarbonate (DMEM-LG, Biowest) supplemented with 0.2% bovine serum albumin (BSA, Sigma Aldrich) and 1% PS. In each well, six pieces of tissue (two from each horse) were cultured in 1.5 mL of growth medium under aseptic and standard conditions in a CO_2_ incubator at 37°C. Specimens harvested from horses affected with EMS were split into control untreated group (Eq SAT_EMS) and experimental group treated with native sex hormone binding globulin (SHBG, Fitzgerald) at a concentration of 50 nM for 24 h (Eq SAT_EMS + SHBG), while SAT obtained from healthy horses were used as a normal control group (Eq SAT_HE). Then all tissue pieces from each group were pulled together, secured in appropriate reagents and subjected to further analysis as described below.

### 2.3 Gene expression analysis

Tissue fragments were lysed in 1 mL of Extrazol (Blirt). Total RNA was isolated from specimens according to the manufacturer’s instructions using the chloroform/phenol method. To verify the quality and quantity of obtained RNA, absorbance at 260 and 280 nm were measured using an Epoch Take3 plate (BioTek). Any remaining DNA from the RNA samples was digested for 30 min at 37°C with DNase I RNase-free (ThermoFisher). DNA-free RNA was used for synthesizing complementary DNA (cDNA) using a PrimeScript RT Reagent Kit (Takara). Both digestion and reverse transcription were performed using a T100 Thermal Cycler (Bio-Rad). Obtained cDNA were used for pre-amplification of determined products using specific primers and annealing temperatures. The pre-amplification mixtures contained both forward and reverse primers at a concentration of 50 mM each (listed in Table 2), 6 ng of cDNA and reagent from SensiFAST SYBR and Fluorescein Kit (Bioline) at 50% of total volume. The pre-amplification based on polymerase chain reaction (PCR) consisted of: initial enzyme activation at 95°C for 2 min, followed by 18 cycles of denaturation at 95°C for 5 s, annealing for 3 min at a temperature dependent on the sequences of primers, and elongation at 72°C for 5 s. The obtained products were diluted in DEPC treated water in a 1:3 ratio and used for Quantitative Reverse-Transcription Polymerase Chain Reaction (RT-qPCR).

**TABLE 2:** Sequences of primers used in RT-qPCR.

Gene	Primers (5′→3′)		Length of amplicon	Accession no.
**AKT1**	**F**	AAG​GAG​ATC​ATG​CAG​CAC​CG	**180**	XM_023628568.1
	**R**	CTC​CAT​CGT​GTC​GTC​TTG​GT		
**CEBPD**	**F**	CTG​TCT​GCC​GAG​AAC​GAG​AA	**186**	XM_023648662.1
	**R**	TCG​GGT​CTG​AGG​TAT​CGG​TC		
**COX4I1**	**F**	GAA​TAG​GGG​CAC​GAA​CGA​GT	**138**	XM_023637444.1
	**R**	GCC​ACC​CAC​TCC​TCT​TCA​AA		
**COX7A1**	**F**	GAA​GAG​GAG​GAC​GCA​GAA​TG	**289**	XM_014733609.2
	**R**	CTG​TTT​CAG​GTC​CTG​TAG​GC		
**COX8A**	**F**	TTC​CCG​ACC​TTG​GGC​TGT​AG	**190**	XM_008541287.1
	**R**	GAG​GTG​AGC​CCA​ATG​GTG​AC		
**CTNNB**	**F**	GAA​CCC​AGC​AGC​AGT​TTG​TG	**219**	NM_001122762.1
	**R**	CAG​CCT​CTT​TGT​CCT​GAG​CA		
**FASN**	**F**	AAA​GGA​GGC​TGC​CCG​AAA​AA	**450**	XM_023651718.1
	**R**	CCA​CAC​GGA​GTC​TCG​TTT​CT		
**GAPDH**	**F**	GAT​GCC​CCA​ATG​TTT​GTG​A	**250**	NM_001163856.1
	**R**	AAG​CAG​GGA​TGA​TGT​TCT​GG		
**GLUT4**	**F**	TTT​GTG​GCA​TTC​TTT​GAG​A	**65**	NM_001081866.2
	**R**	CTGAAGAGCTCAGCCACG		
**IL6**	**F**	CGT​CAC​TCC​AGT​TGC​CTT​CT	**225**	NM_001082496.2
	**R**	GCC​AGT​ACC​TCC​TTG​CTG​TT		
**INSR**	**F**	CCG​TTT​GAG​TCT​GAG​GGG​TC	**254**	XM_023644607.1
	**R**	ACC​GTC​ACA​TTC​CCG​ACA​TC		
**IRF3**	**F**	CCT​ATG​CCC​TCC​ACC​TCT​GA	**124**	XM_001504445.4
	**R**	GGT​ATC​CCT​TGC​CAT​CCA​CG		
**IRS1**	**F**	GGT​GCC​CAA​GGA​CAA​GGA​AGG​A	**276**	XM_023650154.1
	**R**	GAG​AGG​GGG​TGG​CTG​TTG​GAA​A		
**IRS2**	**F**	ATT​CAT​GGT​GGT​CAC​GGG​TC	**197**	XM_023621823.1
	**R**	CCC​TGT​GCG​ATG​GTT​TCT​CT		
**KLF7**	**F**	TAA​AGG​CCC​ACC​AGA​GGA​CT	**253**	XM_003363309.4
	**R**	GTT​TCC​CTC​AGA​CAA​CGG​CT		
**KLF15**	**F**	CGG​GTG​TAC​CAC​ATG​CTG​CCT	**248**	XM_023619835.1
	**R**	TTC​ACA​GAT​GCC​GGT​GCC​CTC		
**KLF2**	**F**	CACACCTGCAGCTACGC	**127**	XM_023625367.1
	**R**	CGAACTTCCAGCCGCAG		
**KLF3**	**F**	GGA​AGA​GAC​CGT​TAC​CTG​TGG	**280**	XM_023638230.1
	**R**	AGG​TGG​TCA​GAG​CGG​GAA​A		
**KLF4**	**F**	GCA​TGT​GCC​CCA​AGA​TCA​AG	**202**	XM_023629843.1
	**R**	GGA​TGA​CAG​TCC​CTG​TTG​CT		
**KLF5**	**F**	GAG​AAA​CGG​CGC​ATC​CAC​T	**275**	XM_023621686.1
	**R**	GCT​CAG​TTC​TGG​TGC​CTC​TTC		
**KLF9**	**F**	TGT​CTG​CGA​AGG​GGA​AAC​AC	**608**	XM_001916945.4
	**R**	GAT​CAT​GCT​GGG​GTG​GAA​CT		
**LPL**	**F**	TCG​CTC​TGA​GGA​CCC​CTA​AA	**297**	XM_005607650.3
	**R**	TGA​TAA​ACC​GGG​CCA​CAT​CC		
**MCP1**	**F**	ATT​GGC​CAA​GGA​GAT​CTG​TG	**167**	NM_001081931.2
	**R**	ATA​TCA​GGG​GGC​ATT​TAG​GG		
**PI3K**	**F**	GAC​TTG​CAC​TTG​GGT​GAC​ATA	**152**	XM_023625590.1
	**R**	TAA​GTT​CCC​GGA​AAG​TCC​CC		
**PLIN1**	**F**	CAA​TGG​CAG​TGA​ACA​AGG​ACC​CG	**120**	XM_023650036.1
	**R**	TTT​TCT​GGA​AGC​ACG​CGC​AGG		
**PNPL2**	**F**	CAT​GGA​ACA​TCT​CGT​TCG​CC	**197**	XM_023654788.1
	**R**	CAC​CTC​GAT​GAT​GTT​GGC​AC		
**PPARA**	**F**	GGC​CTT​CTA​AAC​GTG​GGA​CA	**135**	NM_001242553.1
	**R**	CCG​GAG​GTC​TGC​CAT​TTT​CT		
**PPARGC1A**	**F**	TCTACCTAGGATGCATGG	**93**	XM_014738763.2
	**R**	GTG​CAA​GTA​GAA​ACA​CTG​C		
**PPARGC1B**	**F**	CAA​CTA​TCT​TGC​CGA​CAC​CC	**162**	XM_023617445.1
	**R**	ATG​GGT​TCA​GTC​TCG​GGG​TT		
**PTGES2**	**F**	GGC​CAA​GTA​CAT​GGG​TGC​AG	**334**	XM_023628980.1
	**R**	GCA​TCT​TCC​GTT​GCC​TCT​CG		
**SCD**	**F**	ATG​CTG​ATC​CCC​ACA​ATG​CC	**182**	XM_001500364.4
	**R**	GAA​GCA​CAG​CAA​CAC​GAC​AC		
**SMAD2**	**F**	AGG​GTG​GGG​AGC​AGA​ATA​CC	**89**	XM_023647764.1
	**R**	CCA​ACC​ACT​GTA​GGG​GTC​CA		
**STAT5A**	**F**	AGA​TGC​TGG​CCG​AGG​TCA​AC	**212**	XM_023652507.1
	**R**	AGA​CTT​GGC​CTG​CTG​CTC​AC		

***AKT1***, Protein kinase B; ***CEBPD***, CCAAT/enhancer-binding protein delta; ***COX4I1***, Cytochrome C Oxidase Subunit 4I1; ***COX7A1***, Cytochrome C Oxidase Subunit 7A1; ***COX8A***, Cytochrome C Oxidase Subunit 8A; ***CTNNB***, Catenin Beta; ***EBF1***, Early B cell factor-1; ***FASN***, Fatty Acid Synthase; ***GAPDH***, Glyceraldehyde-3-phosphate dehydrogenase protein; ***GLUT4***, Glucose transporter 4; ***GSK3B***, Glycogen Synthase Kinase 3beta; ***IL6***, Interleukin 6; ***INSR***, Insulin receptor; ***IRF3***, Interferon regulatory factor 3; ***IRS1***, Insulin receptor substrate 1; ***IRS2***, Insulin receptor substrate 1; ***KLF7***, Krueppel-like factor 7; ***KLF15***, Krueppel-like factor 15; ***KLF2***, Krueppel-like factor 2; ***KLF3***, Krueppel-like factor 3; ***KLF4***, Krueppel-like factor 4; ***KLF5***, Krueppel-like factor 5; ***KLF9***, Krueppel-like factor 9; ***LPL***, Lipoprotein lipase; ***MCP1***, Monocyte chemoattractant protein-1; ***PI3K***, Phosphatidylinositol 3-kinase; ***PLIN1***, Perilipin-1; ***PNPL2***, Patatin Like Phospholipase Domain Containing 2; ***PPARA***, Peroxisome proliferator activated receptor alpha; ***PPARGC1A***, Peroxisome proliferator-activated receptor gamma coactivator 1-alpha; ***PPARGC1B***, Peroxisome proliferator-activated receptor gamma coactivator 1-beta; ***PTGES2***, Prostaglandin E Synthase 2; ***SCD***, Stearoyl-CoA desaturase; ***SMAD1***, Mothers against decapentaplegic homolog 1; ***SMAD2***, Mothers against decapentaplegic homolog 2; ***SMAD5***, Mothers against decapentaplegic homolog 5; ***SMAD9***, Mothers against decapentaplegic homolog 9; ***STAT5A***, Signal Transducer And Activator Of Transcription 5A.

For each measurement, 2.5 µL of pre-amplified fragments of cDNA were mixed with forward and reverse primers at concentration of 500 nM each and SensiFAST SYBR and Fluorescein Kit at 50% of reaction total volume. qRT-PCR analysis was performed using a CFX Connect Real-Time PCR Detection System (Bio Rad) under the following thermal conditions: 95°C for 2 min, followed by 40 cycles of: 95°C for 15 s for denaturation, annealing in temperature gradient for 30 s, and extension at 72°C for 15 s with a single fluorescence measurement. The mRNA levels were normalized relative to GAPDH housekeeping gene and the relative gene expression was calculated using the 2^−ΔΔCT^ algorithm.

### 2.4 Protein isolation

Biopsies were collected following 24 h incubation in the presence or absence of SHBG protein, washed with ice-cold PBS and cut with scalpel on ice. Then each 100 mg of tissue was lysed in 500 µL of RIPA buffer containing 1% of protease and phosphatase inhibitor cocktail (Sigma Aldrich). The samples were blended with a mini handheld homogenizer and then incubated for 60 min on ice. Afterwards, the tissues were centrifuged at 12,000 × g, 4°C for 15 min. The upper layer containing fat was discarded and the supernatant was transferred with a syringe into a new tube. To remove excess fat from the sample, the procedure of centrifugation was repeated two times more. Total content of protein was measured spectrophotometrically using the TaKaRa BCA Protein Assay Kit (Takara) and microplate spectrophotometer Epoch (BioTek).

### 2.5 Western blot analysis

The protein samples were mixed with 4× Laemmli Loading Buffer (Bio-Rad) and boiled for 5 min at 95°C. 10 μg of total protein from each specimen was separated by electrophoresis in sodium dodecyl sulphate-polyacrylamide gel at 110 V for 100 min using Mini-PROTEAN Tetra Vertical Electrophoresis Cell (Bio-Rad). Then, the proteins were transferred into previously activated with methanol PVDF membrane (Bio-Rad). The transfer was performed at 100 V for 60 min using the Mini Trans-Blot Cell (Bio-Rad). Afterwards, the membranes were blocked in 5% skimmed milk solution or 5% bovine serum albumin for 2 h at room temperature. After blocking, the membranes were washed once in Tris-buffered saline supplemented with 0.1% Tween-20 detergent (TBST) and then incubated overnight at 4°C with primary antibodies. Washing of membranes with TBST was performed five times for 5 min with TBST and then the membranes were incubated with the secondary antibodies for 2 h at room temperature. After incubation, the membranes were washed again five times for 5 min in TBST before adding the Clarity Western ECL Substrate (Bio-Rad) and visualization in ChemiDoc MP Imaging System (Bio-Rad). The relative adjusted density of protein bands was evaluated using the Image Lab Software (Bio-Rad). To determine the molecular weight of detected proteins, BLUeye Prestained Protein Ladder was used as a marker (Sigma Aldrich). Whole analysis of each protein (listed in Table 3) was performed in at least four repetitions.

**TABLE 3:** Antibodies used in Western blot analysis.

Protein	Manufacturer	Catalog number	Dilution
**FASN**	Affinity Biosciences	DF6106	1:500
**Vinculin**	Sigma Aldrich	V9264	1:8000
**LPL**	Affinity Biosciences	DF12534	1:500
**SCD1**	Affinity Biosciences	DF13253	1:500
**ATGL**	Aviva System Biology	OAEB02425	1:250
**HSL**	Affinity Biosciences	AF6403	1:500
**PLIN1**	Affinity Biosciences	DF7602	1:1000
**p-PI3K**	Biorbyt	orb544410	1:1000
**p-AKT**	Biorbyt	orb304681	1:1000
**GLUT4**	Abcam	ab33780	1:1000
**p-IRS1**	Invitrogen	PA1-1054	1:660
**p-IRS2**	Affinity Biosciences	AF8383	1:1000
**SHBG**	Biorbyt	orb11366	1:500
**p-IR**	Affinity Biosciences	AF3099	1:500
**ERK1/2**	Affinity Biosciences	AF1055	1:500
**PDIA3**	Aviva System Biology	ARP63565	1:250
**Anti-mouse IgG, HRP conjugated**	Jackson ImmunoResearch	115-035-146	1:8000
**Anti-rabbit IgG, HRP conjugated**	Sigma Aldrich	AP156P	1:2500
**Anti-goat IgG, HRP conjugated**	Jackson ImmunoResearch	805-035-180	1:8000

***FASN***, Fatty Acid Synthase; ***LPL***, Lipoprotein lipase; ***SCD1***, Stearoyl-CoA desaturase-1; ***ATGL***, Adipose triglyceride lipase; ***HSL***, Hormone-sensitive lipase; ***PLIN1***, Perilipin 1; ***p-PI3K***, Phosphorylated Phosphoinositide-3-kinase; ***p-AKT***, Phosphorylated Protein kinase B; ***GLUT4***, Glucose transporter 4; ***p-IRS1***, Phosphorylated Insulin receptor substrate 1; ***p-IRS2***, Phosphorylated Insulin receptor substrate 2; ***SHBG***, Sex hormone-binding globulin; ***p-IR***, Phosphorylated Insulin receptor; ***GSK3B***, Glycogen Synthase Kinase 3 Beta; ***ERK1/2***, extracellular signal-regulated protein kinase 1/2; ***PDIA3***, Protein disulfide isomerase associated 3; ***HRP***, Horseradish peroxidase.

### 2.6 Enzyme-linked immunosorbent assay

Concentrations of inflammatory factors (IL6, TNFA, PGE2, MCP1) in tissues were evaluated by enzyme linked immunosorbent assays (ELISA) in accordance with the manufacturers’ instructions (IL6, TNFA and MCP1—BTLAB, PGE2—EIAab). For each assay, 7 µg of total protein solution were served per well as an antigen. Concentration of each marker was measured in four technical repetitions using microplate spectrophotometer Epoch (BioTek).

### 2.7 Lipidomics profiling

#### 2.7.1 Extraction of FAs from subcutaneous adipose tissue specimens

The extraction of lipid metabolites was performed with Folsh method from 500 mg of SAT (Folch et al., 1957). Briefly, 2 mL of a chloroform: methanol (2:1) solution was added to each sample, and subjected to vigorous shaking for 2 h. Thereafter, the organic solvents were separated and the process was repeated. Then, the extraction solvent from particular samples were combined and evaporated with a rotary-vacuum evaporator. All samples were prepared in three repetitions.

#### 2.7.2 Preparation of fatty acid methyl esters (FAMEs)

At the first step, 50 µg of C17:0 fatty acid (Sigma-Aldrich, Steinheim, Germany) was added as an internal standard (IS) to each sample. Then, the samples were hydrolyzed with 2 mL 0.5 M solution of potassium hydroxide (KOH) (POCH, Gliwice, Poland) in methanol (POCH, Gliwice, Poland) by boiling the flask content for 10 min under the reflux column. Then, after cooling, 1.5 mL of 14% (v/v) solution of boron trifluoride (BF3) in methanol (Sigma-Aldrich, Steinheim, Germany) was added and the esterification process was carried out for 10 min at 80°C under the reflux column. After cooling, 1 mL of saturated solution of sodium chloride (NaCl) (POCH, Gliwice, Poland) was added and the FAMEs were extracted with two portions, 1.5 mL each, of hexane (POCH, Gliwice, Poland). After the extraction, the hexane phases from particular samples were combined and dried above the anhydrous magnesium(II) sulphate(VI) (MgSO_4_) (POCH, Gliwice, Poland). Thereafter, the samples were concentrated to approx. 200 μL with rotary-vacuum evaporator and transferred to chromatographical vials equipped with glass inserts.

#### 2.7.3 Qualitative and quantitative analysis of the FAMEs profiles

For GC-MS analyses, a Shimadzu GCMS-QP2020 (Shimadzu, Kyoto, Japan) apparatus equipped with a ZB-FAME column (Phenomenex, Torrance, CA, United States) (60 m × 0.25 mm i. d. × 0.25 µm layer thickness) was used. 1 μL of each sample was injected at 80°C in split mode 2, and helium (1.8 mL·min^−1^) was used as a carrier gaz. The GC thermal program was as follows: 80°C held for 2 min, then raised to 180°C at a rate of 3°C·min^−1^, then to 240°C at a rate of 8°C·min^−1^ and held for 4 min. The MS operational conditions were as follows: interface temperature 240°C; ion source temperature 220°C; scanning mode 40–400 m/z.

The identification of FAMEs present in the samples was based on the reference analysis of Supelco 37 Component FAME Mix (Sigma-Aldrich, Steinheim, Germany). The quantification of the FAMes present in the samples was based on the amount of added IS.

### 2.8 Statistical analysis and bioinformatics

The data were presented as mean ± standard error of the mean (SEM). One-way analyses of variance (ANOVA) were conducted using GraphPad Prism 8 software (GraphPad Software, La Jolla, CA, United States) with a Tukey’s correction. Differences with probability of *p* < 0.05 were indicated with an asterisk (*), those with *p* < 0.001 were marked with two asterisks (**), differences with *p* < 0.001 were represented with three asterisks (***), and differences with *p* < 0.0001 were showed with four asterisks (****).

Integrated peak areas are normalized by internal standard amount. The average and standard deviation for each individual molecular species is calculated. The data discrimination analysis using SIMCA-17 (Umetrics, Sweden) group-based models were constructed using 3D principal component analysis (PCA) and 2D partial least squares-discriminant analysis (PLS-DA) to identify differentially abundant fatty acids between groups. The loading plot of PCA was obtained to identify the lipids negative and positive association to the principal components (PCs). The most important fatty acids identified by PLS-DA model for control, EMS and SHBG-treated EMS groups were selected based on values of the variable importance in projection (VIP) plot score. Pathway enrichment and topology analysis was performed on the 13 assigned fatty acids. All matched pathways were plotted based on their *p*-value obtained from pathway enrichment analysis and pathway impact score. The significance of each pathway was indicated by a color gradient, with yellow representing higher *p*-values and red representing lower *p*-values. The pathway impact score was reflected in the size of the circle, with a larger circle indicating a higher impact score (Xia et al., 2015). Moreover, the pathways were also used to confirm their significance with *p* <0.05 and FDR≤0.05 by using the MetaboAnalyst software (http://www.metaboanalyst.ca/).

## 3 Results

### 3.1 SHBG normalizes the lipidome profile of EMS adipose tissue

**FIGURE 1:**
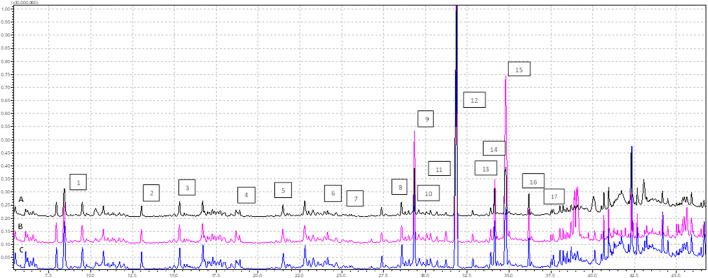
Profile of FAMEs identified in the tissues samples (analysis time 7.5 min—37.0 min), where **(A)** EMS tissue, **(B)** EMS + SHBG, **(C)** CRTL; 1—C8:0, 2—C10:0, 3—C11:0, 4—C12:0, 5—C13:0, 6—C14:0, 7—C14:1n5, 8—C15:1n5, 9—C16:0, 10—C16:1n7, 11—C17:0, 12—C17:1, 13—C18:0, 14—C18:1n9 (E), 15—C18:1n9 (Z), 16—C18:2n7, 17—C18:3n3.

**FIGURE 2:**
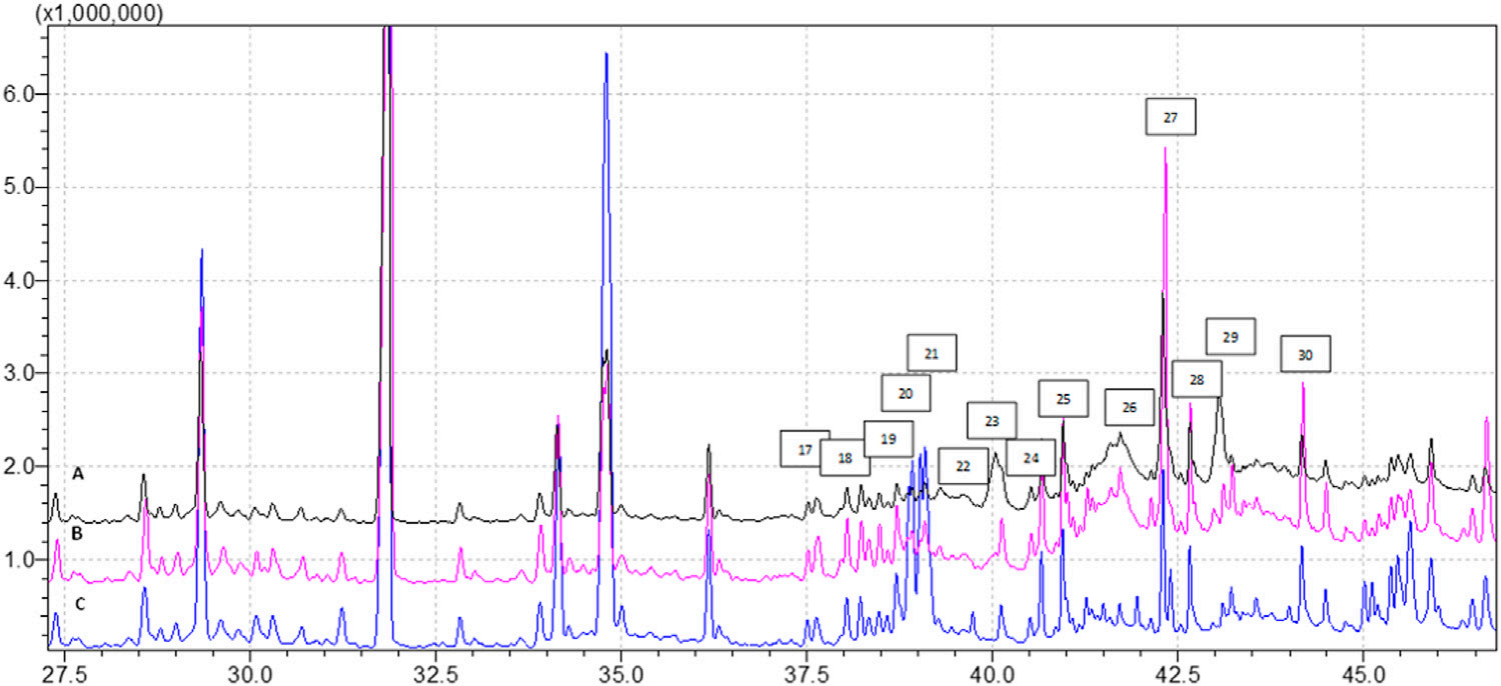
Profile of FAMEs identified in the tissues samples (analysis time 37.5 min—45.0 min), where **(A)** EMS tissue, **(B)** EMS + SHBG, **(C)** CRTL; 17—C18:3n3, 18—C20:0, 19—C20:1n9, 20—C20:2n6, 21—C21:0, 22—C20:3n6, 23—C20:4n6, 24—C22:0, 25—C20:5n3, 26—C22:2n6, 27—C24:0, 28—C25:1, 29—C24:1, 30—C22:6n3.

We conducted GC-MS based lipidomics analysis on subcutaneous adipose tissue samples from a total of nine subjects, including control, EMS, and SHBG-treated EMS groups. A total of 25 fatty acids were successfully identified in the tissue samples (Figures 1, 2; Table 4).

**TABLE 4:** Fatty acids composition of SAT-derived metabolites identified by GCMS- based lipidomics from control, EMS and EMS tissue with SHBG treatment samples.

Fatty acids (FAMEs)	Names	CTRL (*n* = 3) mean ± SD	EMS (*n* = 3) mean ± SD	SHBG (*n* = 3) mean ± SD	VIP value^a^
		[μg/500 mg tissue]			
**Me20:3n3**	8,11,14-Eicosatrienoic acid	0.0210 ± 0.000	0.0040 ± 0.0040	0.0060 ± 0.0010	**1.7530**
**Me 18:2n6, Z,Z**	Linoelaidic acid	1.7040 ± 0.1130	1.206 ± 0.0270	1.132 ± 0.2140	**1.6801**
**Me20:1n9**	Eicosenoic acid	0.0857 ± 0.0075	0.7493 ± 0.1595	0.2050 ± 0.0450	**1.2001**
**Me 18:3n3**	Alpha-Linolenic acid	0.4343 ± 0.0205	3.125 ± 0.6600	0.6997 ± 0.0675	**1.1327**
**Me 16:1n7**	Palmitolinoleic acid	0.4103 ± 0.0045	0.3990 ± 0.0440	0.2860 ± 0.0950	**1.1033**
**Me 18:2n6, E,E**	Linoleic acid	0.0030 ± 0.0030	0.0300 ± 0.0130	0.0037 ± 0.0035	**1.0128**
**Me18:0**	FAHFA(18:0/9-O-18:0)	8.128 ± 0.2935	10.27 ± 0.7750	4.759 ± 0.4335	0.9871
**18:1n9,Z**	Oleic acid	8.0280 ± 2.8080	31.56 ± 12.7800	6.245 ± 2.7290	0.9611
**Me 14:0**	Methyl tetradecanoate	1.6410 ± 0.272	1.787 ± 0.4075	1.030 ± 0.1215	0.9605
**Me 11:0**	Undecylenic acid	0.5803 ± 0.0385	1.855 ± 0.2410	0.2150 ± 0.0580	0.9342
**Me 21:0**	Heneicosanoic acid	0.1667 ± 0.0395	0.2093 ± 0.0405	0.0827 ± 0.0055	0.9316
**Me 17:1, Z**	(Z)-9-Heptadecenoic acid	0.0477 ± 0.0055	0.05367 ± 0.0295	0.0227 ± 0.0055	0.9262
**Me 16:0**	Methyl hexadecanoic acid	16.0900 ± 0.3765	24.94 ± 0.8955	10.35 ± 0.2405	0.9164
**Me 15:0**	Pentadecanoic acid	0.5597 ± 0.0165	0.7233 ± 0.0935	0.3937 ± 0.0275	0.9101
**Me 12:0**	Methyl dodecanoate	1.8430 ± 0.0585	3.892 ± 0.3670	0.7983 ± 0.2545	0.8962
**Me 8:0**	Caprylic acid	0.3220 ± 0.0720	0.4497 ± 0.03750	0.1743 ± 0.0465	0.8861
**Me 10:0**	Capric acid	0.4220 ± 0.0500	0.5453 ± 0.1245	0.2410 ± 0.0840	0.8645
**Me 23:0**	Tricosanoic acid	0.3037 ± 0.0685	0.4823 ± 0.0775	0.2250 ± 0.0580	0.8342
**Me 18:1n9,E**	Elaidic acid	0.0553 ± 0.0095	0.1460 ± 0.0830	0.0157 ± 0.0065	0.8179
**Me 24:0**	Tetracosanoic acid	1.7460 ± 0.4050	2.377 ± 0.3320	1.440 ± 0.4490	0.7945
**Me 22:0**	Behenic acid	1.9900 ± 0.4385	2.551 ± 0.6925	1.343 ± 0.4070	0.7684
**Me 22:1n10**	Erucic acid	0.8557 ± 0.0785	0.8787 ± 0.1375	0.7060 ± 0.2520	0.7582
**Me 20:0**	Arachidonic acid	1.2640 ± 0.3895	1.862 ± 0.4120	1.014 ± 0.1035	0.7481
**Me 20:2n6**	Eicosadienoic acid	0.8557 ± 0.0785	0.8787 ± 0.1375	0.7060 ± 0.2520	0.7228
**Me 22:2n6**	Docosadienoate (22:2n6)	0.0130 ± 0.0020	0.0103 ± 0.0105	0.0160 ± 0.0030	0.6862

^a^ Variable importance in projection value from PLS-DA, model.

***Me***, Methyl esters; ***FAMEs***, Fatty acid methyl esters; The numerical symbol: total amount of (C)arbon atoms of the fatty acid, and the number of (D)ouble (unsaturated) bonds.

To explore the changes in fatty acid composition, we generated 3D PCA and loading plots of the fatty acid dataset and built an OPLS-DA model to predict the total scores of the groups. The models were then validated and assessed using R^2^X (cum), the predictive and orthogonal variation in X, R^2^Y (cum), the total sum of variation in Y explained, and Q^2^ (cum) the goodness of prediction, calculated by significant cross-validated predictive residuals (CV-ANOVA) *p*-values for the comparisons between the control, EMS, and SHBG groups (Table 4).

**FIGURE 3:**
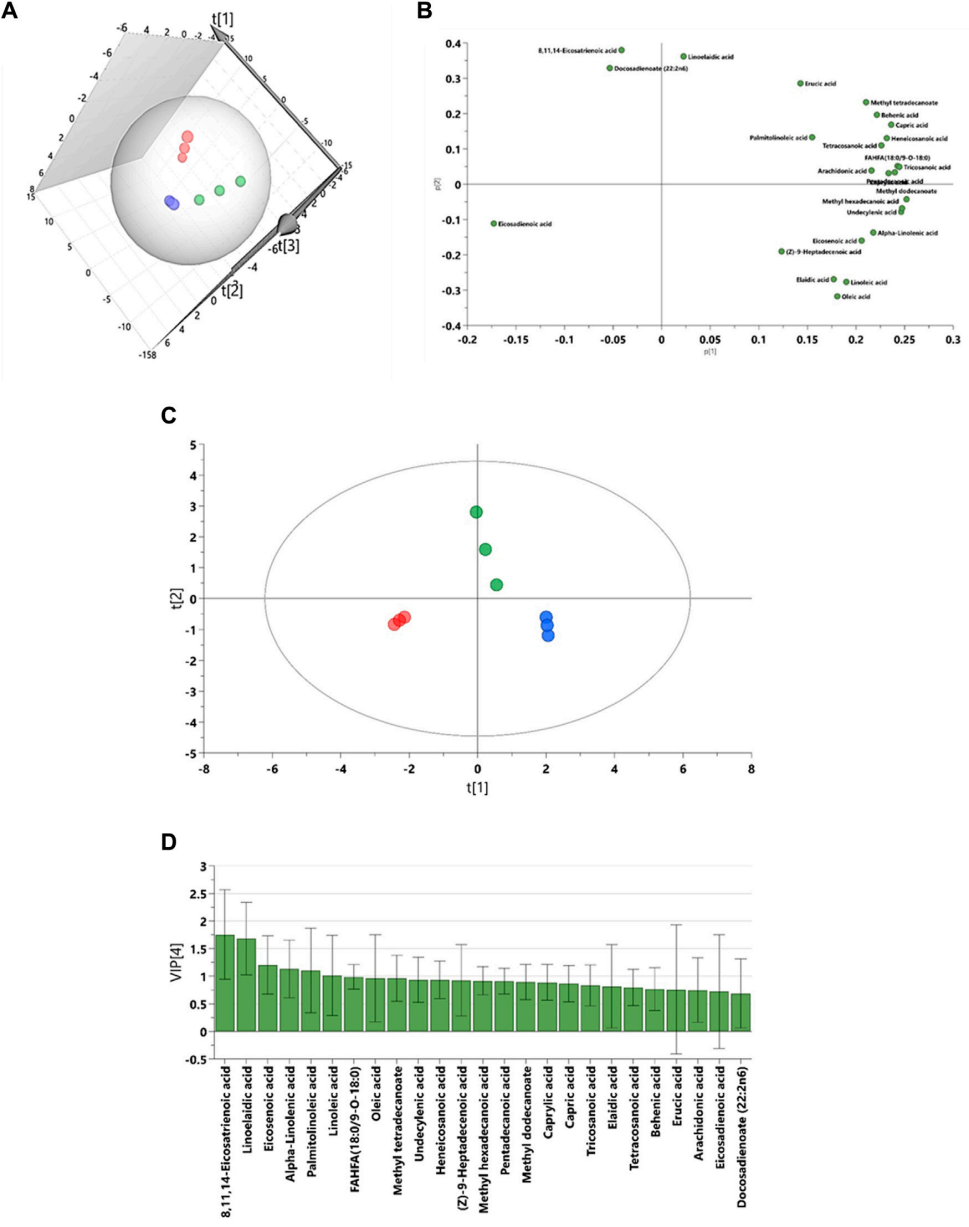
Graphical representation for lipidomic data driven models for subcutaneous adipose tissue. **(A)** The 3D principal component analysis (PCA) scores plot. **(B)** The partial least squares projections to latent structures discriminant analysis (PLS-DA). **(C)** The loadings plots for PCA model. **(D)** The variable importance in projection (VIP) score plot from PLS-DA model. Blue: control SAT (CTRL); Red: EMS SAT; Green: SHBG-treated EMS SAT.

The results from SAT fatty acids profiling showed that 3D PCA model has a good separation between control, EMS and SHBG-treated EMS groups (Figure 3A). The PLS-DA model was rebuilt from variable importance in projection (VIP) score plot with VIP value above 1 and validated with statistical parameters [R^2^X (cum), R^2^Y (cum) and Q^2^] by using just two PLS components and the cross-validated residuals (CV-ANOVA) of PLS-R model was significant (Figure 3B; Table 5). Moreover, the PCA-loading plot showed the fatty acids have negative contribution to PC1 including 8,11,14-eicosatrienoic acid, docosadienoate and eicosadienoic acid. However, the remaining fatty acids have positive contribution to PC1 (Figure 3C). The 6 fatty acids with VIP (variable importance in projection) value above 1.00 were selected including 8,11,14-eicosatrienoic acid, linoelaidic acid, eicosenoic acid, alpha-Linolenic acid, palmitolinoleic acid and linoleic acid (Figure 3D).

**TABLE 5:** The multivariate analysis model summary of adipose tissue extract GCMS- based lipidomic in comparisons between control, EMS and SHBG treatment.

Group comparison	Model type	PC/LV	N =	R^2^X (cum)	R^2^Y (cum)	Q^2^ (cum)	CV-ANOVA *p*-value
**CTRL vs. EMS vs. EMS_SHBG**	PCA-X	4	9	0.976	−	0.873	−
**CTRL vs. EMS vs. EMS_SHBG**	PLS-DA VIP>1	2	9	0.906	0.894	0.772	**1.24 × 10** ^**−2**^

***PCA-X***, Principal component analysis; ***PLS-DA***, Partial least squares-discriminant analysis; ***VIP***, Variable importance in projection; ***LV***, Latent variable; ***N***, Number of samples. ***R^2^X(cum)/R^2^Y(cum)***, Cumulative explained variation; ***Q^2^(cum)***, Goodness of prediction; ***CV-ANOVA***, Cross-validated predictive residuals of a model.

**FIGURE 4:**
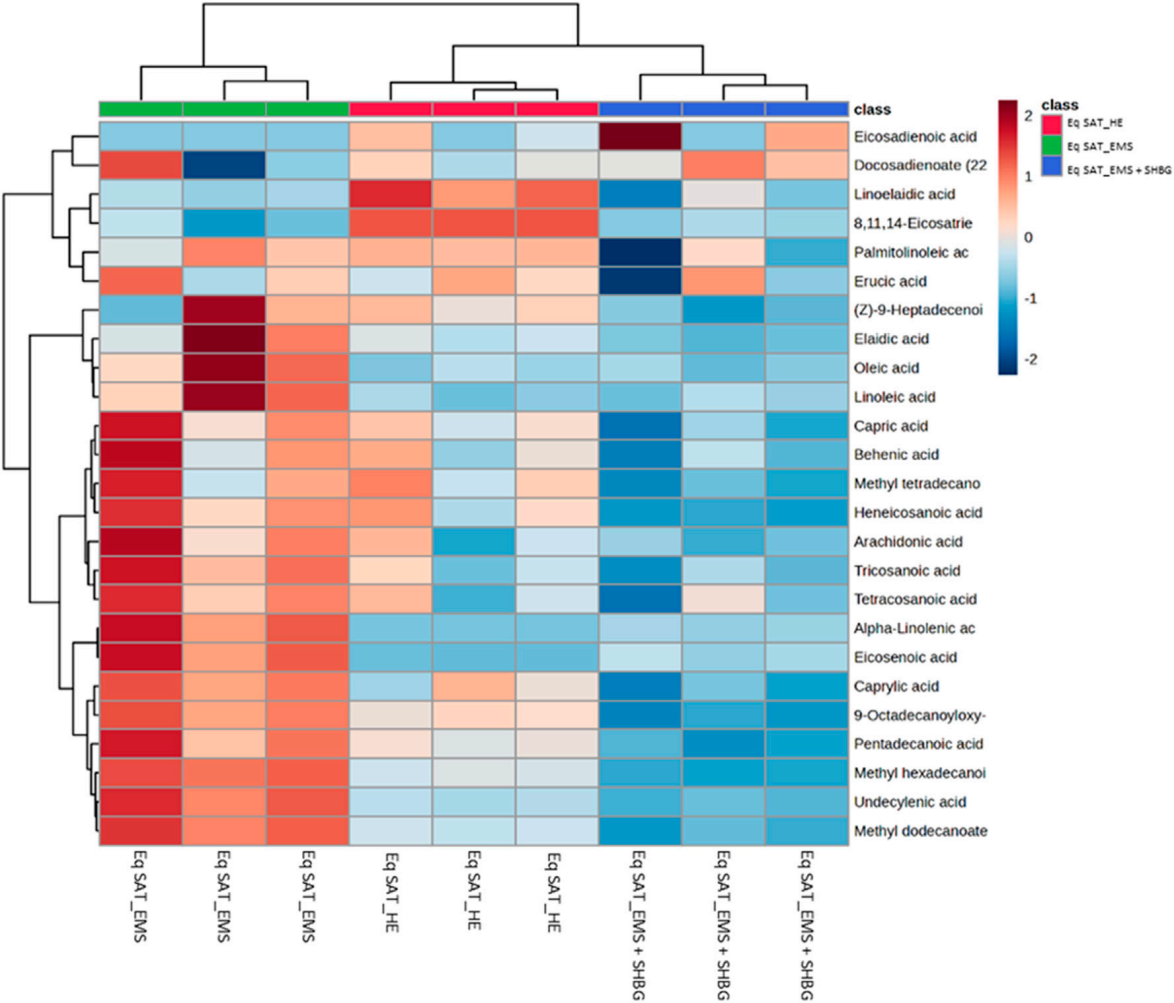
Hierarchical clustering heatmap analysis of fatty acids in equine subcutaneous adipose tissue. Each colored cell on the map corresponds to a concentration value. The top 25 features from the Eq SAT_HE, Eq SAT_EMS and Eq SAT_EMS + SHBG groups are presented and ranked by t-tests to retain the most contrasting patterns. Values are measured by Euclidean distance with a Ward clustering algorithm (*n* = 3 per group). *p ≤ 0.05 for each comparison. Red color indicates high level, and blue color indicates low level.

We performed hierarchical clustering analysis using Euclidean distance and Ward D. linkage to identify any scattered samples. The resulting heatmap revealed distinct variations in the fatty acids among the control SAT, EMS SAT, and the SHBG-treated EMS SAT groups (Figure 4). The fatty acids were clearly grouped based on different percentages between groups. For example, 8,11,14-eicosatrienoic acid and linoelaidic acid were abundant in the control group but not in the EMS and EMS treated with SHBG groups. Conversely, Oleic acid, Linoleic acid, Heneicosanoic acid, Arachidonic acid, Tricosanoic acid, Tetracosanoic acid, Alpha-Linolenic acid, Eicosenoic acid, Caprylic acid, FAHFA (18:0/9-O-18:0) (9-hexadecanoyloxy-octadecanoic acid), Pentadecanoic acid, Methyl hexadecanoic acid, Undecylenic acid and Methyl dodecanoate were significantly more abundant in the EMS group but not in SHBG-treated EMS SAT.

**FIGURE 5:**
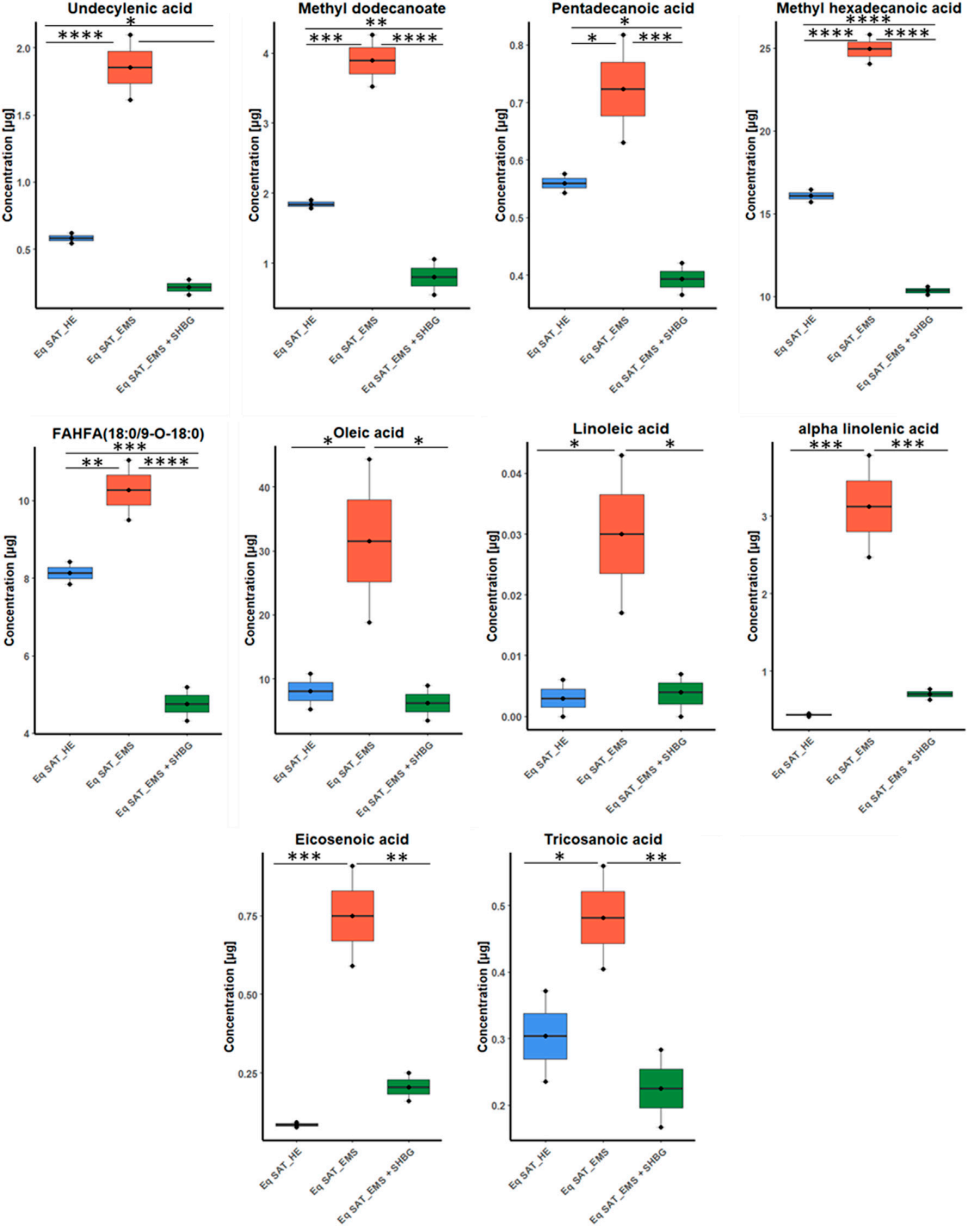
Boxplots for fatty acids sets showed efficacy of SHBG treatment, and after *p*-value adjustment. Whiskers—1.5 × interquartile range (IQR); bar—median; box—range between first quartile (Q1) and third quartile (Q3). Black points—data points. *Adjusted *p*-value <0.05.

The assigned fatty acids were tested by ANOVA, and showed different tendency between groups. For instance, ten fatty acids have downregulated by 55.5% from 18 significant fatty acids after SHBG treatment, with significant differences observed between the Eq SAT_EMS and Eq SAT_EMS + SHBG groups. These results suggest that the negative changes in the fatty acid profiles induced by EMS are reversible and can be effectively normalized by SHBG treatment such as undecylenic acid, methyl dodecanoate, pentadecanoic acid, methyl hexadecanoic acid, FAHFA (18:0/9-O-18:0), oleic acid, linoleic acid, alpha-Linolenic acid, eicosenoic acid and tricosanoic acid (Figure 5).

**FIGURE 6:**
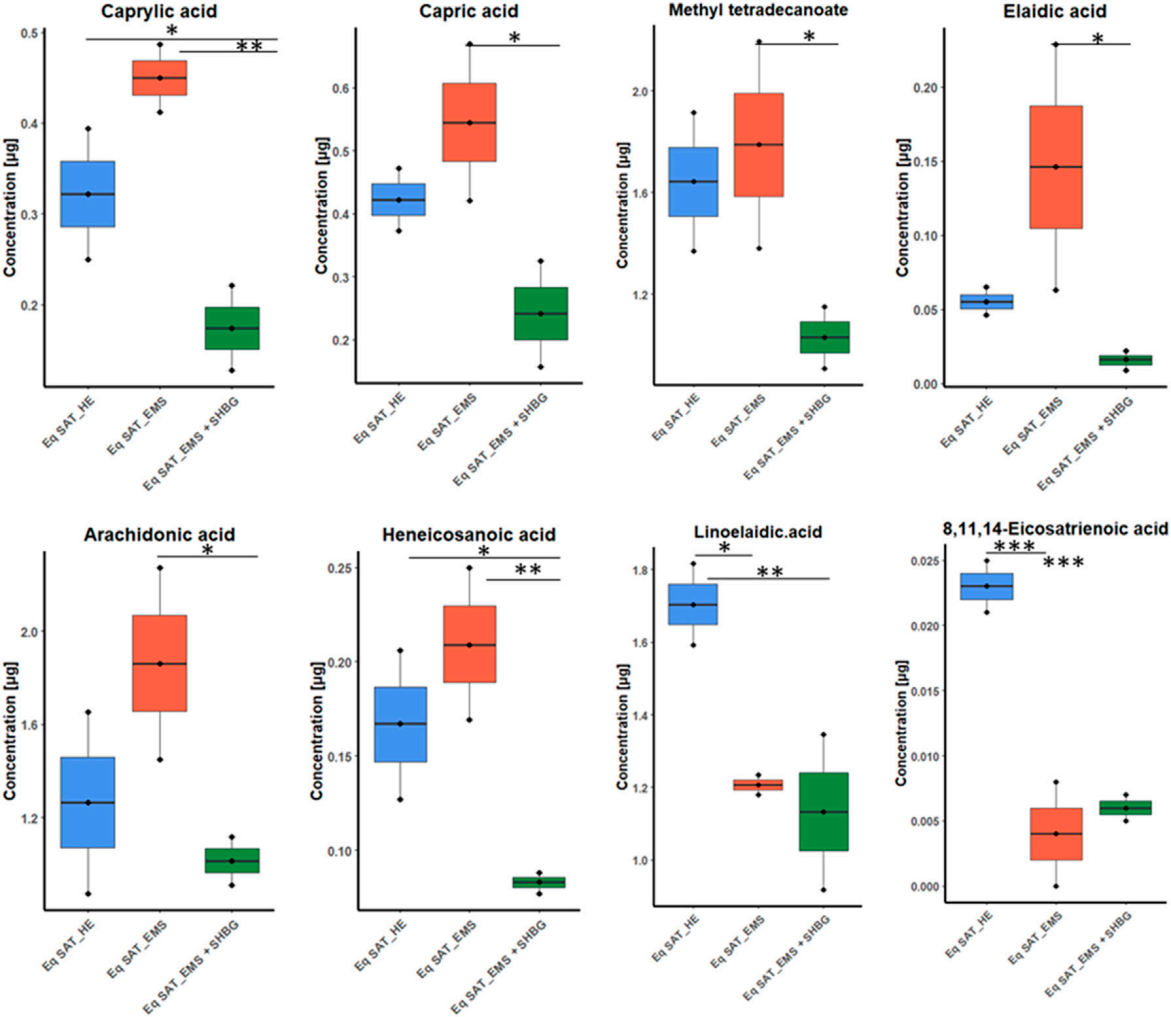
Boxplots for the ANOVA significant fatty acids showing different tendency between groups, and after *p*-value adjustment. Whiskers—1.5 × interquartile range (IQR); bar—median; box—range between first quartile (Q1) and third quartile (Q3). Black points—data points. *Adjusted *p*-value <0.05.

Moreover, from ANOVA test we found six fatty acids have moderate differences between control and EMS groups including caprylic acid, capric acid, methyl tetradecanoate, elaidic acid, arachidonic acid and heneicosanoic acid. Additionally, only two fatty acids showed no significant differences between EMS and SHBG-treated groups such as linoelaidic acid and 8,11,14-eicosatrienoic acid (Figure 6).

**FIGURE 7:**
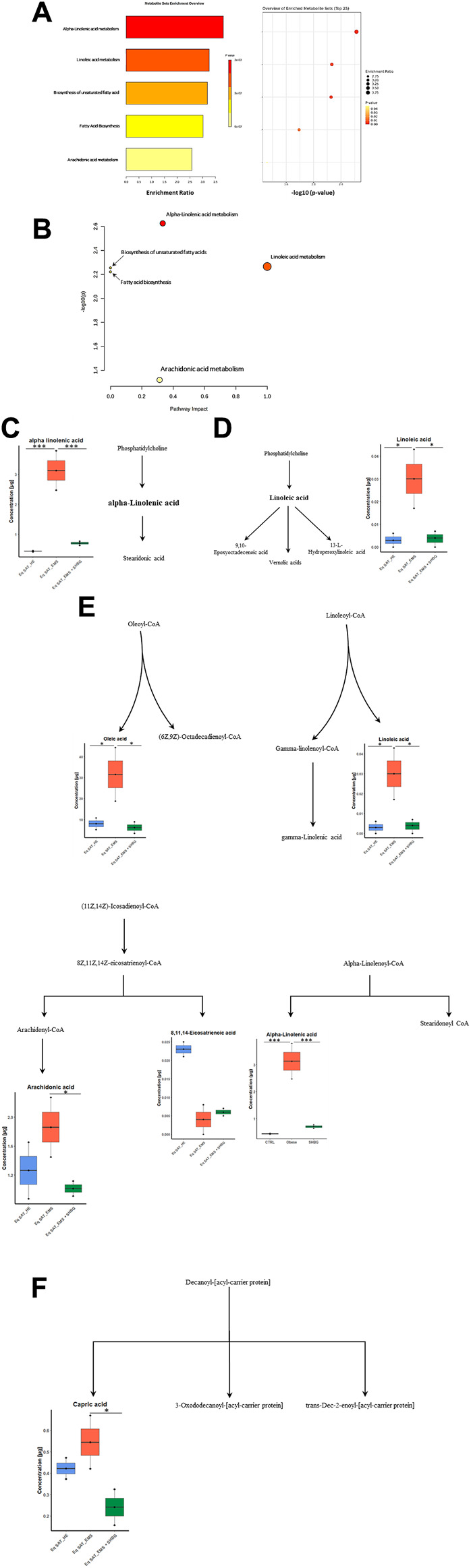
Graphical representations for GC-MS dataset driven models of adipose tissue lipidome. **(A)** Summary plot for Quantitative Enrichment Analysis (QEA). **(B)** Pathway enrichment and topology analysis was performed on the 13 identified metabolites. All matched pathways were plotted based on their *p*-value obtained from pathway enrichment analysis and pathway impact score. The significance of each pathway was indicated by a color gradient, with yellow representing higher *p*-values and red representing lower *p*-values. The pathway impact score was reflected in the size of the circle, with a larger circle indicating a higher impact score. **(C–F)** Altered fatty acids pathway to SHBG treatment with KEGG ID from the merged dataset KEGG and SMPDB reference pathways and interaction networks were generated in Metaboanalyst. **(C)** Alpha-Linolenic acid metabolism. **(D)** Linoleic acid metabolism. **(E)** Biosynthesis of unsaturated fatty acids. **(F)** Fatty acid biosynthesis. Bold text: detected fatty acids; control SAT (CTRL); Red: EMS SAT; Green: SHBG-treated EMS SAT.

We conducted pathway and enrichment analysis of the 25 identified fatty acids using Metaboanalyst 4.0, and the associated metabolic pathways are presented in Figure 7. We calculated the pathway impact value based on pathway topology analysis and identified potential target pathways. Alpha-Linolenic acid metabolism, Linoleic acid metabolism, biosynthesis of unsaturated fatty acids, and Arachidonic acid metabolism (Table 6; Figures 7A–F) were the primary fatty acid pathways affected by SHBG treatment.

**TABLE 6:** Summary of pathways analysis of fatty acids dataset.

Pathway name	Match status	*p*-Value	−log(p)	FDR	Impact
Alpha-Linolenic acid metabolism	1/13	0.002	2.626	0.008	0.333
Linoleic acid metabolism	1/5	0.005	2.266	0.008	1.000
Biosynthesis of unsaturated fatty acids	5/36	0.006	2.255	0.008	0.000
Fatty acid biosynthesis	2/47	0.006	2.221	0.008	0.000
Arachidonic acid metabolism	1/36	0.048	1.320	0.048	0.314

### 3.2 SHBG modulates lipids metabolism-related mediators in EMS SAT

**FIGURE 8:**
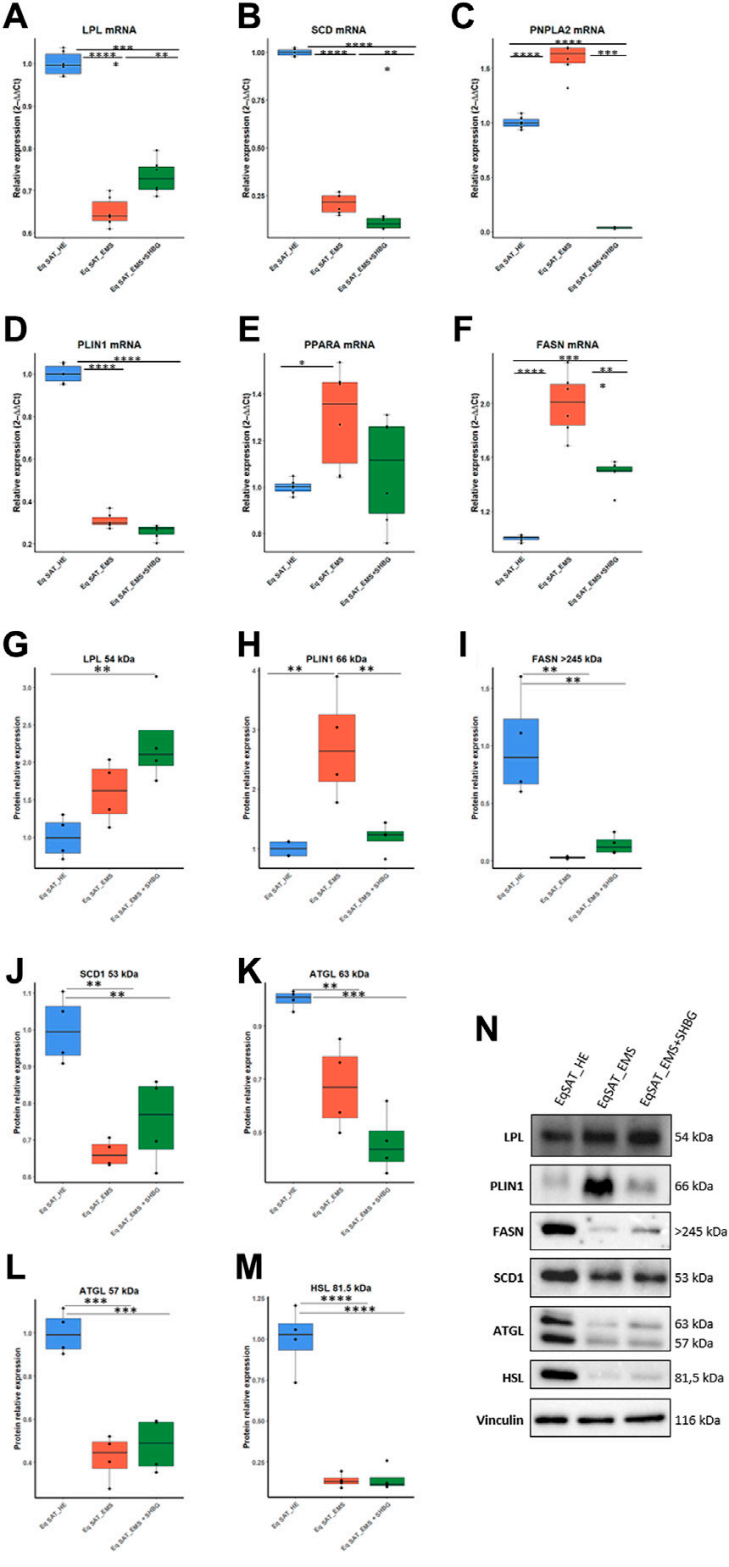
Influence of SHBG treatment of lipid metabolism related modulators interplay in SAT (A–N). The relative expression levels of LPL **(A)**, SCD **(B)**, PNPLA2 **(C)**, PLIN1 **(D)**, PPARA **(E)**, FASN **(F)** in SAT were analyzed by RT-PCR. Protein levels of LPL **(G)**, PLIN1 **(H)**, FASN **(I)**, SCD1 **(J)**, ATGL **(K, L)** and HSL **(M)** were analyzed by Western blot (**(N)**, representative membranes). Representative data are shown as mean ± SD. ** *p*-value <0.01, *** *p*-value<0.001 and **** *p*-value<0.0001.

To investigate whether SHBG is involved in the regulation of lipid metabolism in equine SAT, the expression of mediators involved in the regulation of this process was analyzed at the mRNA and protein level. The obtained data showed that the relative expression of LPL in EMS SAT was significantly downregulated compared to control tissue (Figure 8A; *p* < 0.0001), which has been subsequently upregulated following application of SHBG for 24 h (Figure 8A; *p* < 0.01). Relative expression of SCD in EMS SAT was drastically decreased when compared to the control tissue (Figure 8B; *p* < 0.0001) and remained unaffected after the SHBG treatment compared to the control (*p* < 0.0001) and EMS SAT (Figure 8B; *p* < 0.001). In contrast to these results, expression of PNPLA2 was significantly upregulated compared to the EMS group (Figure 8C; *p* < 0.0001), while exogenous SHBG treatment drastically decrease its expression level when compared to control and EMS tissue (Figure 8C; *p* < 0.0001). Relative expression of PLIN1 was downregulated in SAT from EMS horses (Figure 8D; *p* < 0.0001) and similar tendency was observed after exposure to SHBG (Figure 8D). mRNA level of PPARA in EMS tissue was increased compared to control (Figure 8E; *p < 05*), and SHBG rescue enabled the restoration of normal PPARA expression levels (Figure 8E). Transcript level of FASN was significantly increased in SAT of EMS horses compared to the control healthy group (Figure 8F; *p* < 0.0001), and abrogated following SHBG treatment (Figure 8F; *p* < 0.0001). Basic regulators of lipids metabolism have been further analyzed at the protein level. EMS adipose tissue was characterized by comparable LPL protein level in regards to the control group (Figures 8G, N), however, the treatment of EMS tissue with exogenous SHBG resulted in a remarkable upregulation of the protein when we compared to the control tissue (Figure 8G). Similar to mRNA, PLIN1 protein level was upregulated in EMS group compared to the control (Figures 8H, N; *p* < 0.01). Exogenous SHBG treatment further decreased its expression to a level comparable to that of control tissue (Figure 8H; *p* < 0.01). In contrast to these results, FASN protein appeared markedly decreased in EMS SAT compared to control group (Figures 8H, N; *p* < 0.0001). Treatment of EMS SAT with exogenous SHBG induced slight augmentation of FASN protein level however of statistical insignificance compared to both control (*p* < 0.01) and EMS SAT (*p* < 0.0001). Significantly downregulated of SCD1 (Figures 8J, N; *p* < 0.01), ATGL (Figures 8K, L, N; *p* < 0.01 for 63 kDa subunit, *p* < 0.001 for 57 kDa subunit), and HSL (Figures 8M, N; *p* < 0.0001) protein levels were also observed in EMS group in opposition to the control tissue. Moreover, application of exogenous SHBG induced a moderate elevation of SCD1 protein (Figure 8J), and the 57 kDa ATGL isoform (Figure 8L), while HSL level remained unchanged (Figure 8M).

### 3.3 SHBG promotes the expression of adipogenesis negative regulators in EMS SAT

**FIGURE 9:**
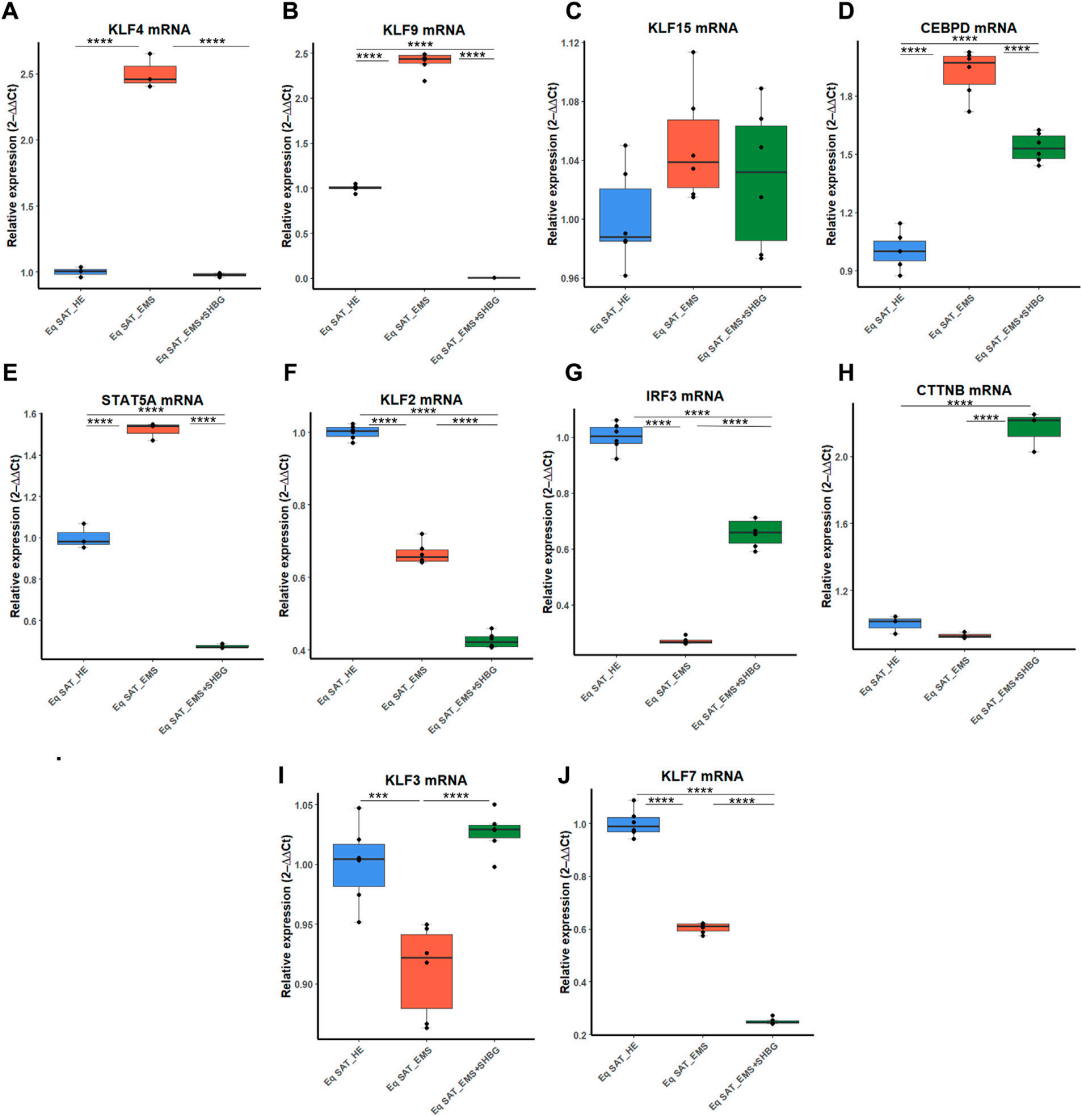
Representative graphs showing the relative expression levels of promotors (A–J) and inhibitors (K–P) of adipogenesis. The expression analysis of KLF4 **(A)**, KLF9 **(B)**, KLF15 **(C)**, CEBPD **(D)**, STAT5A **(E)**, KLF2 **(F)**, IRF3 **(G)**, CTNNB **(H)**, KLF3 **(I)**, KLF7 **(J)** was performed by RT-PCR tool. Representative data are shown as mean ± SD. *p-value < 0.05, ** p-value < 0.01, *** *p*-value < 0.001, ****p-value < 0.0001.

To better investigate the relation between SHBG protein and adipose tissue turnover, an analysis of the expression of adipogenesis promotors (Figures 9A–J) and inhibitors (Figures 9H–K) was performed using RT-qPCR technique. At first, expression levels of kruppel-like factor family (KLF), promotors of adipogenesis were analyzed. The obtained results showed that KLF4 (Figure 9A) and KLF9 (Figure 9B) expression levels were significantly increased in EMS SAT compared to the control group (*p* < 0.0001). After treatment of the tissue with exogenous SHBG, the mRNA levels of these promoters were all downregulated. The level of KLF4 was emphatically reduced when compared to EMS SAT (Figure 9B; *p* < 000.1) and appeared at a similar level compared to control cells (Figure 9A). Similarly, treatment of EMS fat tissue with exogenous SHBG drastically reduced KLF9 levels relative to EMS group (Figure 9B; *p* < 0.0001), and compared to control (Figure 9B; *p* < 0.0001). However, no differences were observed in the KLF15 transcript level, both in EMS SAT and after treatment with exogenous SHBG compared to control group (Figure 9C). Obtained results additionally showed that in EMS SAT, the expression level of the pro-adipogenic CEBPD was drastically upregulated compared to control group (Figure 9D; *p* < 0.0001), which has been critically lowered upon SHBG treatment (Figure 9D; *p* < 0.0001). Similar levels were observed for STAT5A mRNA expression, that was found significantly activated in EMS-derived SAT compared to the control group (Figure 9F; *p* < 0.0001), and subsequently mitigated following SHBG application (Figure 9E; *p* < 0.0001).

Next, the expression patterns of factors negatively regulating adipogenesis were analyzed. The obtained data showed a critical loss in adipogenesis regulatory signals in EMS animals. The relative expression level of KLF2 and KLF7 was significantly downregulated in EMS tissue compared to control one (Figures 9F, J; *p* < 0.0001), and interestingly, further treatment of the tissues with SHBG reduced their expression when compared to EMS group (Figures 9F, J; *p* < 0.0001) and control group (Figures 9F, J; *p* < 0.0001). Next, the relative expression level of KLF3 was also found to be significantly downregulated in EMS SAT compared to control (Figure 9I; *p* < 0.001), however, subsequent treatment of EMS tissue with exogenous SHBG protein exerted a noticeable augmented expression of KLF3 by opposition to EMS group (Figure 9I; *p* < 0.0001) and was normalized to an equivalent level of that in control SAT (Figure 9I). In the case of the IRF3 inhibitor, mRNA level was similarly downregulated in EMS group when compared to the control tissues (Figure 9G; *p* < 0.0001). Exogenous SHBG application induced a visible increase in IRF3 level in relation to EMS SAT (Figure 9G; *p* < 0.0001), however it remained moderately lower than that observed in control SAT (Figure 9G; *p* < 0.0001). Analysis of CTNNB expression level showed no differences between control and EMS groups (Figure 9H). However, in EMS SAT treated with exogenous SHBG protein, a significant upregulation in the relative mRNA level of this gene was observed compared to both control (Figure 9H; *p* < 0.0001) and EMS tissues (Figure 9H; *p* < 0.0001).

### 3.4 SHBG enhances the expression of mitochondrial metabolism markers in SAT

**FIGURE 10:**
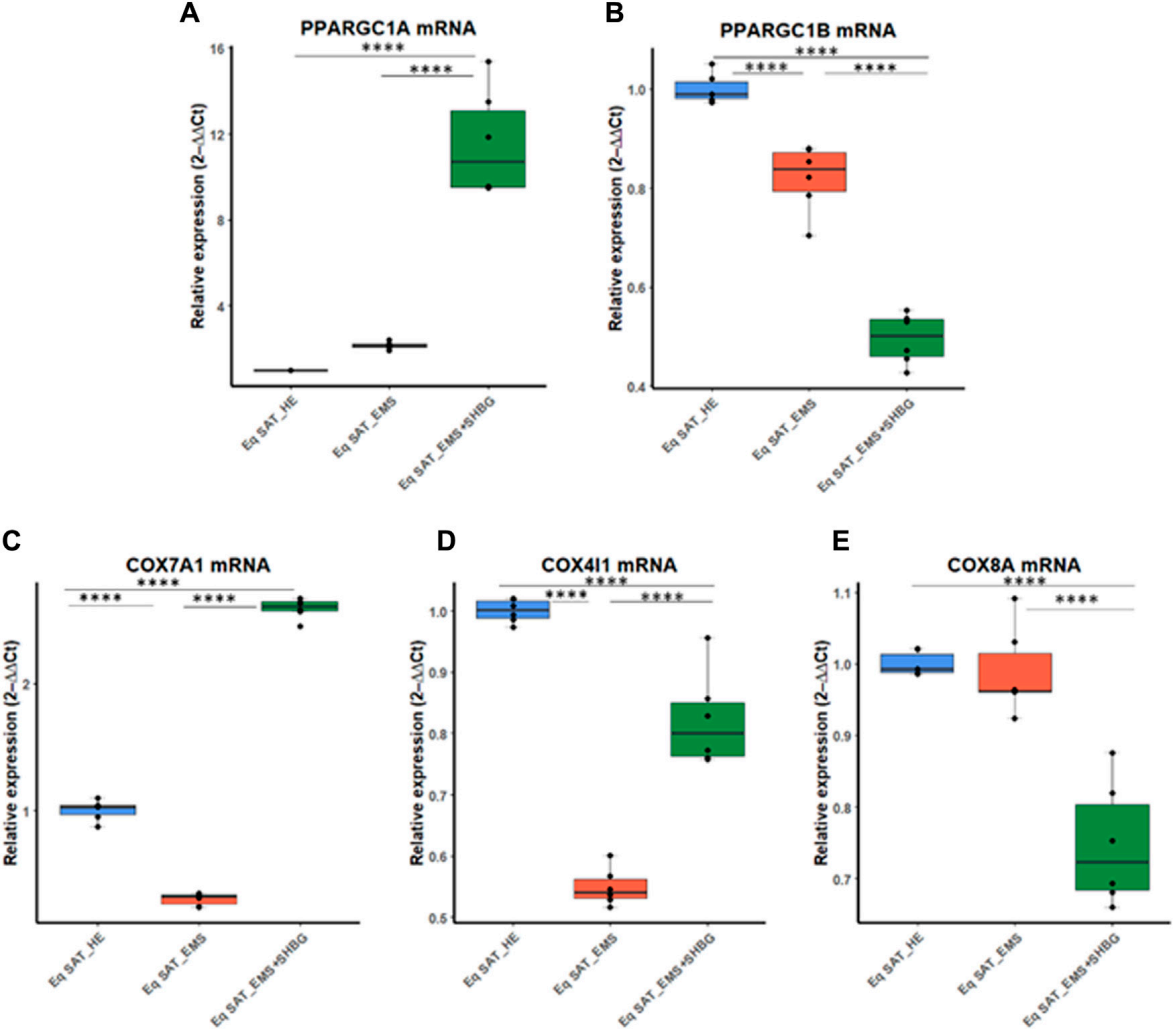
SHBG and mitochondrial metabolism markers interplay analysis. The relative expression levels of PPARGC1A **(A)**, PPARGC1B **(B)**, COX7A1 **(C)**, COX4I1 **(D)**, and COX8A **(E)** were analyzed by RT-PCR. Representative data are shown as mean ± SD. **** p-value < 0.0001.

To better investigate the relationship between SHBG protein and mitochondrial functions in the course of adipocytes metabolism, the main markers involved in the mitochondrial oxidative phosphorylation machinery have been analyzed at the mRNA level. The obtained results showed that the relative expression level of PPARGC1A remained unchanged in EMS tissue (Figure 10A), however, appeared elevated in SAT treated with exogenous SHBG, both relative to control (Figure 10A; *p* < 0.0001) and EMS fat (Figure 10A; *p* < 0.0001). On the other hand, EMS SAT had significantly downregulated PPARGC1B expression compared to the control group (Figure 2B; *p* < 0.0001). Next, the expression of mitochondrial cytochrome c oxidase (COX) family members was analyzed. The obtained results showed that the relative expression level of COX7A1 was significantly downregulated in EMS SAT (Figure 10C; *p* < 0.0001), and SHBG protein intervention drastically increased the mRNA level of this gene so that it was significantly higher compared to both control (Figure 10C; *p* < 0.0001) and EMS tissue (Figure 10C; *p* <0.0001). A similar trend was observed for the COX4I1 expression level. While it appeared significantly downregulated in EMS fat (Figure 10D; *p* < 0.0001), exogenous SHBG application resulted in a substantial augmentation of its transcription rate compared to the control (Figure 10D; *p* < 0.0001) and EMS group (Figure 10D; *p* < 0.0001). A difference in expression levels between control cells and EMS SAT was not observed for COX8A expression (Figure 10E). However, treatment of EMS fat with exogenous SHBG reduced its mRNA levels relative to control (Figure 10E; *p* < 0.0001) and EMS tissue (Figure E10; *p* < 0.0001).

### 3.5 SHBG restores insulin signalling in EMS SAT

**FIGURE 11:**
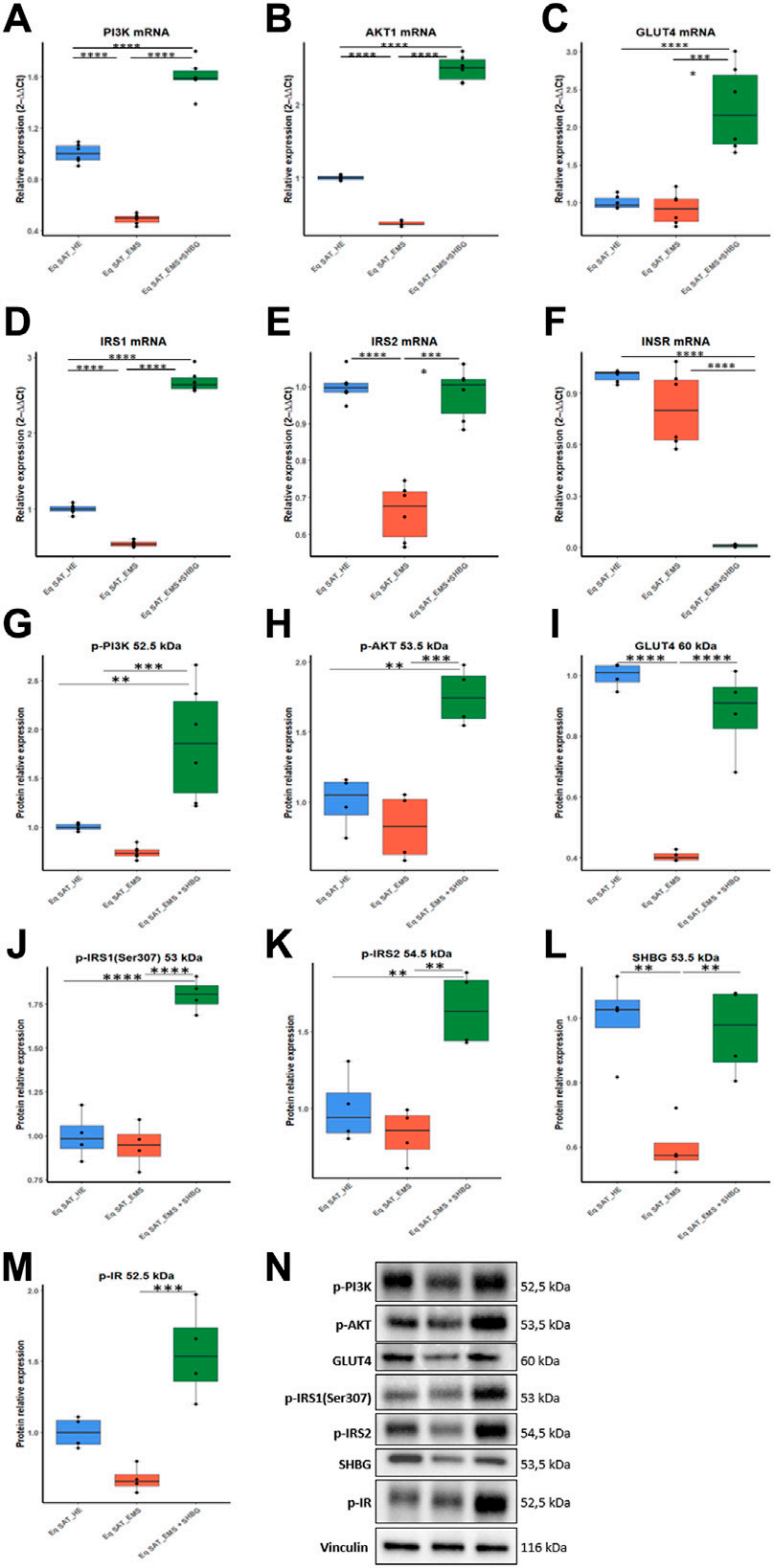
Involvement of SHBG in the insulin signaling pathway. Relative expression levels of PI3K **(A)**, AKT1 **(B)**, GLUT4 **(C)**, IRS1 **(D)**, IRS2 **(E)**, INSR **(F)** were analyzed by qRT-PCR. Protein levels of PI3K **(G)**, AKT **(H)**, GLUT4 **(I)**, IRS1 **(J)**, IRS2 **(K)**, SHBG **(L)**, IR **(M)** were analyzed by Western Blot. Representative immunoblots **(N)**. Representative data are shown as mean ± SD. ** *p*-value < 0.01, *** *p*-value < 0.001 and **** *p*-value < 0.0001.

To thoroughly investigate the role of the SHBG protein in insulin signaling transduction, the gene expression and phosphorylation rates of the main factors involved in insulin cascade were investigated. EMS SAT displayed critical loss in expression efficiency of key insulin signaling mediators including PI3K (Figure 11A; *p* < 0.0001), AKT (Figure 11B; *p* < 0.0001) and IRS1/2 (Figures 11D, E; *p* < 0.0001). Nevertheless, expression levels of INSR and GLUT4 were found unaffected by the EMS condition (Figures 11C, F). Interestingly, SHBG intervention enabled to substantially enhance the transcription of PI3K, AKT, GLUT4 and IRS1/2 when compared to EMS untreated tissues as well as healthy controls.

To seek on the potential influence of SHBG on insulin-mediated phosphorylation cascades, Western blot analysis has been used for proteins profiling. Obtained data highlighted the profound defects in phosphorylation mechanisms underlying insulin signaling of EMS SAT. both phospho-Pi3K, -AKT and -IR levels were found slightly lowered under EMS condition when compared to control tissues (Figures 11G, H, M). Notably, EMS SAT also displayed a critical suppressed GLUT4 protein level (Figure 11I), evoking a reduced capacity for glucose uptake. Accordingly, treatment of EMS SAT with 50 nM SHBG exerted a potent insulin sensitizing effect by restoring proper phosphorylation levels of Pi3K, AKT, IR and IRS1/2 and by augmenting the expression level of GLUT4 protein in relation to EMS untreated group. Particularly, SHBG induced a stimulation of insulin-induced phosphorylation of its transducers at a higher level than the threshold observed for control tissue, suggesting its great potential as an insulin sensitizer agent.

Another interesting finding lies in the observed loss in endogenous SHBG protein in EMS SAT compared to healthy tissue (Figures 11L, P; *p* < 0.01), which confirms the critical importance of SHBG as a metabolic mediator.

### 3.5 SHBG mitigates inflammatory pathways by inhibiting the PDIA3/ERK axis

**FIGURE 12:**
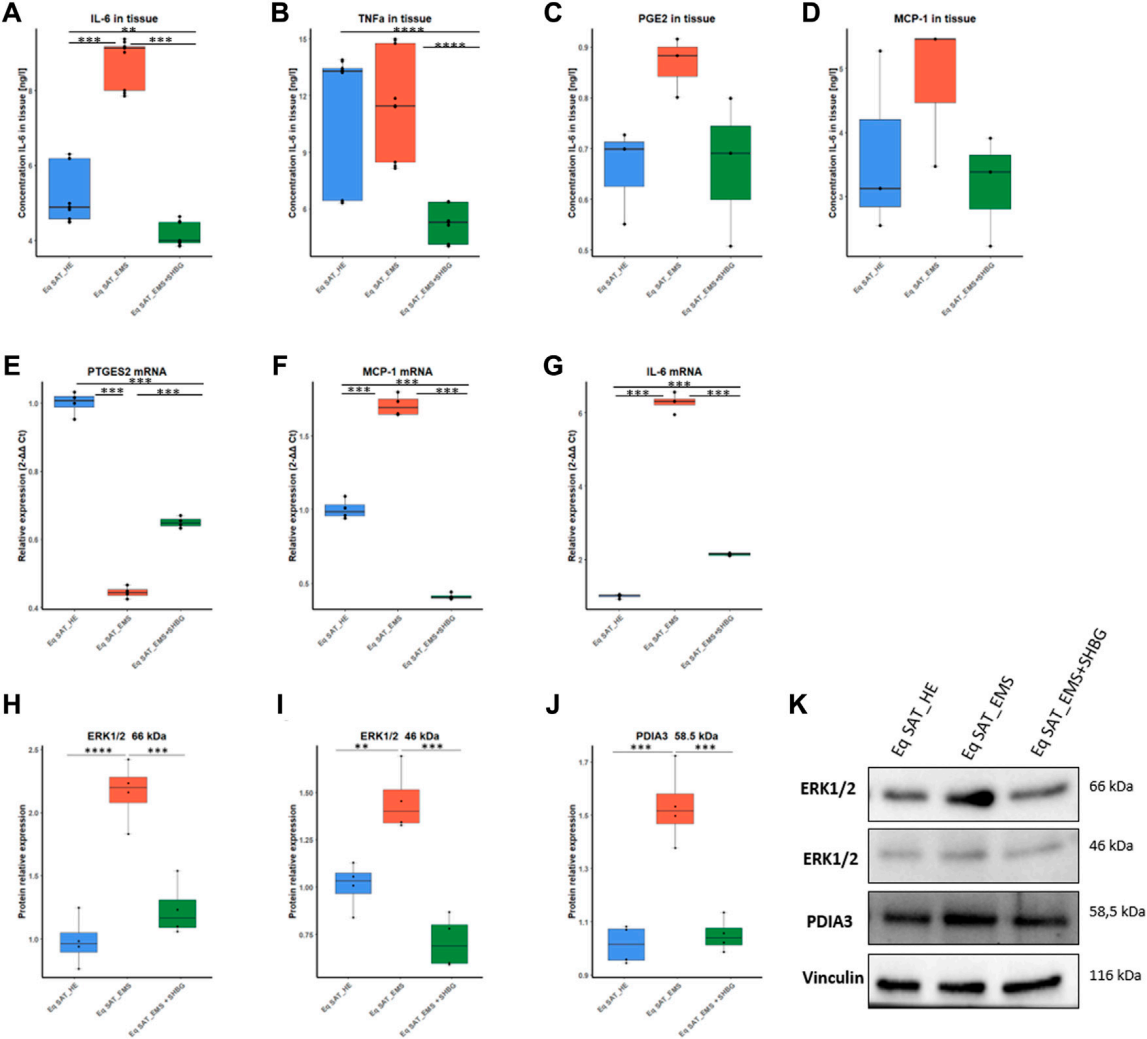
Involvement of SHBG in the inflammation pathway. Graphs showing the concentration of IL-6 **(A)**, TNF **(B)**, PGE2 **(C)**, and MCP-1 **(D)** in SAT analysed by ELISA tests. Relative expression levels of PTGES2 **(E)**, MCP-1 **(F)**, and IL-6 **(G)** were analysed by qRT-PCR. Protein levels of ERK1/2 **(H,I)** and PDIA3 **(J)** were analyzed by Western Blot. Representative blot membranes **(K)**. Representative data are shown as mean ± SD. ** *p*-value < 0.01, *** *p*-value < 0.001 and **** *p*-value < 0.0001.

To investigate the effect of SHBG protein on inflammation in SAT, the levels of selected pro-inflammatory cytokines were evaluated at the tissue level using ELISA assays (Figures 12A–D). The obtained results showed that the concentration of IL-6 in EMS tissue was significantly higher compared to normal tissue (Figure 12A; *p* < 0.001), which has been substantially lowered following SHBG treatment application (Figure 12A; *p* < 0.001). Next, TNF-α has been evaluated at comparable levels in both control and EMS SAT (Figure 12B), and surprisingly, SHBG treatment further decreases TNF-α concentration in the EMS SAT compared to untreated EMS fat (Figure 12B; *p* < 0.001) and also control SAT (Figure 12B; *p* < 0.001). MCP-1 (Figure 12C) and PGE2 (Figure 12D) concentrations were found slightly increased in EMS SAT and lowered after SHBG intervention, however with statistical insignificance.

In order to further elucidate the possible molecular pathways, put in play of anti-inflammatory potential of SHBG, changes in PDIA3/ERK proteins have been tested. Western blots analysis showed that in EMS SAT, the level of ERK1 66 kDa protein was significantly increased when compared to control fat (Figures 12H, K; *p* < 0.0001), while in EMS tissue treated with exogenous SHBG resulted in a visible downregulated protein compared to untreated tissue (Figures 12H, K; *p* < 0.001), and reached comparable level to that of control SAT (Figures 12H, K). A similar tendency was noted for ERK2 46 kDa isoform, where EMS SAT exhibited higher ERK2 abundance compared to control (Figures 12I, K; *p* < 0.01), while exogenous SHBG protein addition triggered a sharp decrease in ERK2 amount with respect to EMS tissue (Figures 12I, K; *p* < 0.001) and was found furthermore normalized to control basal level (Figures 12I, K). Interestingly, an important augmented expression of PDIA3 protein was noted in EMS-derived adipose tissue by opposition to control group (Figures 12J, K; *p* < 0.001). Here also, SHBG intervention efficiently regulated and normalized the levels of PDIA3 protein in EMS SAT in connection to both EMS untreated group (Figure 12J; *p* < 0.001) and healthy fat tissue (Figures 12J, K), suggesting that SHBG might attenuate inflammatory responses via the inhibition of the PDIA3/ERK pathway.

## 4 Discussion

Adipose tissue is a fat storage and endocrine organ that plays a pivotal role in lipids and glucose homeostasis. The release of different adipokines including adiponectin, leptin, visfatin, resistin, tumor necrosis factor-α (TNF-α), and monocyte chemoattractant protein-1 (MCP-1) regulate and mediate the fate of both residing cells and non-adipogenic organs such as liver and pancreas (Jung and Choi, 2014). Many lines of evidence clearly indicated the implication of adipose tissue disease and malfunction in the development and progression of endocrine and inflammatory disorders including insulin resistance, making it an essential target for therapeutics. While EMS affected horses display profound altered adipose tissue metabolism and dysregulated lipids turnover, the loss in SHBG protein has been previously correlated with impaired insulin signalling and general metabolic disruption in the course of insulin resistance, systemic inflammation, obesity and metabolic syndrome (Reynolds et al., 2019; Bourebaba et al., 2022). Therefore, the impact of SHBG treatment on EMS subcutaneous adipose tissue (SAT) metabolic dynamics has been investigated. Obtained data demonstrated that *ex-vivo* SHBG application restores insulin sensitivity, regulates lipids metabolism programs and reduces inflammation in fat tissue biopsies derived from EMS affected horses.

The choice of SHBG as a therapeutic target was motivated by the existence of a certain number of reports having demonstrated a direct interrelation between the abnormal expansion and dysfunction of adipose tissue, whether visceral or subcutaneous, and the drop in SHBG levels. De Simone and others (De Simone et al., 2001), have thus found that massive visceral adipose tissue accumulation strongly associates with elevated circulating insulin levels and negatively correlates with serum SHBG level in obese adolescents. As well, Nielsen et al. (2007), demonstrated that excessive SAT was closely associated with declined SHBG levels in obese men patients. Likewise, Kim and collaborators (Kim et al., 2017), found that changes in both VAT and SAT were inversely related to changes in SHBG, and that reduction in both VAT and SAT results in higher levels of SHBG in both men and women with diabetes, suggesting SHBG as a valuable candidate for obesity-associates complications intervention.

Adipose tissue dysfunction is regarded as a major pathophysiologic component of metabolic syndrome. Besides releasing a large amount of reactive adipokines, malfunctioning adipose tissue strongly alters the lipid metabolism and turnover machinery. The untargeted lipidome analysis of EMS-derived adipose tissue evidenced a profound alteration in the lipidic profile with abnormal elevated levels of saturated (SFAs), polyunsaturated (PUFAs), trans and cis unsaturated fatty acids, as well as fatty acid hydroxy fatty acids (FAHFAs). Specifically, EMS SAT exhibited significant higher levels of palmitic, lauric, oleic, α-linoleic, elaidic, capric and caprylic acids. Excessive saturated and trans unsaturated fats intake has been previously evidenced as a major contributor of obesity and related diseases development (De Souza et al., 2015; Cena and Calder, 2020). The SFAs thus induce fundamental molecular changes that impair the metabolic capacity of cells. Weijers (2015), for example, reported that increased SFAs levels participates in insulin resistance and glucose intolerance onset as they induce an increase in membrane stiffness and a partial loss in glucose transporters (GLUTs) capacity. Moreover, SFAs such as palmitic acid (16:0) or lauric acid (12:0) can also elicit inflammatory responses by antagonizing the TLR4/NFκB/JNK/ERK axis, which subsequently alters the phosphorylation of the insulin receptor, leading to adipose tissue insulin resistance (Huang et al., 2012). In terms of the cis unsaturated oleic acid (18:1n9,Z), Malodobra-Mazur et al. (2019), showed in their research that oleic acid surplus induces excessive expression of leptin, lipids accumulation, adiponectin suppression and simultaneous insulin signalling and glucose utilization impairment in murine adipocytes; as far as Xie and co (Xie et al., 2006), who reported that high oleic acid levels trigger insulin resistance in adipocytes via the inhibition of Akt phosphorylation and Glut4 translocation. Adipose tissue malfunction and low-grade inflammation are associated to lipid metabolism disruption and increased circulating and ectopic PUFA levels. A critical increase in delta-6 and -9 desaturases activities and reduction in delta-5 desaturase have been linked to reduced capacity to convert α-linoleic acid into EPA and DHA (ω-3 PUFA) pro-resolving mediators in overweight/obese subjects, suggesting a possible decreased rate of α-linoleic acid conversion to beneficial ω-3 PUFA in altered adipose tissue (Takic et al., 2022). In line with the observed lipids imbalance in EMS SAT, SHBG treatment showed remarkable ability to normalize the levels of the detected fatty acids by lowering the abundance of FAHFA, palmitic, lauric, oleic, elaidic, capric and caprylic acids. Modified SHBG levels has already been strongly associated with *de novo* lipogenesis alterations, increased pro-inflammatory dietary lipids intake and fatty acids subclasses imbalance (Wang et al., 2015; Simons et al., 2021; Liu et al., 2022). Accordingly, Bataille and others (Bataille et al., 2005), showcased SHBG as a central determinant in the regulation of lipids profile, in relation to is sex hormones bindings capacity and its significant association with insulin. Here, we showed for the first time the clear influence of SHBG on individual adipose tissue FFAs abundance under EMS condition, and bring the evidence for a possible regulatory effect of SHBG on lipid metabolism.

To further illustrate the SHBG implication in adipose tissue lipid shift, the expression levels of key enzymes involved in lipolysis and lipogenesis has been investigated. EMS adipose tissue displayed significant dysregulated lipid metabolism master mediators with evidence of either transcriptional or posttranslational alterations. Indeed, a critical loss in lipoprotein lipase (LPL) and stearoyl CoA desaturase 1 (SCD1), has been observed at both mRNA and protein levels, while increased mRNA expression of fatty acid synthase (FASN), and adipose triglyceride lipase—ATGL (PNPLA2) were found to oppositely correspond to lower proteins levels. Conversely, the expression of perilipin-1 (PLN1) appeared upregulated at the protein level only. Previous investigations reported on the association of disrupted lipid metabolism and lipolysis mediators with metabolic disorders development. Lower FASN protein levels, a central lipogenesis enzyme that catalyses *de novo* saturated FA biosynthesis, has been associated with hyperglycaemia, insulin resistance and obesity (Mayas et al., 2010). Likewise, depleted expression of LPL, a rate-limiting enzyme regulating TG-FFAs absorption and storage has been observed in adipose tissue of obese, diabetic, atherosclerotic and dyslipidaemic patients (Kursawe et al., 2010; Serra et al., 2017). Interestingly, contradictory findings regarding SCD1 implication in metabolic syndrome development have demonstrated that while overactivation of SCD1 enzyme triggers insulin resistance and increased fat depots in rodents, SCD1 expression is inversely correlated with inflammation and insulin resistance in human patients (Peter et al., 2009; Gong et al., 2011), which strongly suggest that in horses, SCD1 loss is a hallmark of EMS and SAT metabolic instability. Moreover, elevated perilipin-1 (PLN1) protein abundance, a key lipolysis modulator has been found similarly to our observations in obese human subjects, which has been proposed as a compensatory mechanism limiting basal lipolysis (Kern et al., 2004). By contrast, triglyceride lipase (ATGL) deficiency has been reported to deteriorate the adipose tissue metabolic microenvironment, and consequently participate in obesity and hyperlipidaemia occurrence (Lai et al., 2022). Lipolysis plays a crucial role in maintaining lipid homeostasis and its alteration has been shown to trigger adipocyte hypertrophy and severe metabolic inflexibility (Li et al., 2022). Our obtained data evidenced the pro-lipolytic potential of SHBG by the selective perilipin 1 modulation. In white adipose tissue, perilipin 1 restricts the access of cytosolic lipases to adipocytes lipid droplets to promote the storage of triacylglycerols (Sztalryd and Brasaemle, 2017). Treatment of EMS SAT with exogenous SHBG resulted in a substantial downregulation of perilipin 1 protein to a basal level. These observations are in contrast to a previous study showing that SHBG overexpression stimulates PLIN mRNA and protein expression in a model of human SHBG transgenic mice fed with HFD. However, the same study evidenced the ability of SHBG to activate lipolytic pathways and to prevent adipocytes hypertrophy by restoring HSL protein levels (Saez-Lopez et al., 2020). In the same vein, the response of human visceral adipocytes to SHBG treatment were in agreement with observed effects on mouse white adipose tissue, where a visible activation of HSL and ERK-1/2 phosphorylation have been observed, indicating that SHBG similarly promotes lipolysis in human mature visceral adipocytes (Saez-Lopez et al., 2020). These findings are in opposition to our obtained data demonstrating no significant regulatory effect of SHBG toward HSL and ATGL lipases expression. Such discrepancies maybe explained by the variation in the studied models and the already observed species differences of the regulatory mechanisms governing lipid turnover in rodents and horses (Breidenbach et al., 1998). Moreover, the absence of lipolysis stimuli in our *ex-vivo* model may explain the non-observable activation of lipases in EMS SAT, and obtained data could provide evidence of EMS-associated resistance to basal lipolysis mediated by increased expression of the gatekeeper perilipin which is reversed by SHBG treatment. In point of fact, PLIN1 deletion has been shown to minimize weight gain in HFD db/db mice and proposed as an obesity preventive mediator. Therewith, high expression of PLIN1 in fibroblasts resulted in a strong suppression of basal lipolytic rates (Tansey et al., 2004), suggesting its critical role in aberrant fats accumulation and storage, as well as the potential role of SHBG in limiting PLIN1 bioavailability in adipocytes. However, further investigation of stimulated lipolytic pathways is necessary to elucidate whether SHBG can promote the PLIN1 phosphorylation and HSL activation. Little is known regarding lipolytic pathways in equines, and to our best knowledge, this is the first study analysing the implication of SHBG in the modulation of lipid metabolism under EMS condition. Previous report showed that EMS adipose tissue is characterized by adipocytes hypertrophy and lipids engorgements, evoking defects in lipolysis as demonstrated in the present study (Reynolds et al., 2019). Other findings indicated on the loss in SHBG protein in the course of insulin resistance and metabolic syndrome (De Oya et al., 2009; Winters et al., 2014), which stays in line with the present investigation, where a marked SHBG protein downregulation has been observed in EMS SAT biopsies SHBG. Hence, the incubation of EMS SAT with SHBG protein induced a downregulation of the FASN gene expression and increased mRNA and protein levels of LPL. These results are in accordance with previous investigation demonstrating the suppressive effect of SHBG toward FASN transcript and the potential anti-lipogenic effect of SHBG which has been postulated to be at least partly related to its ability to increase intracellular cAMP (Yamazaki et al., 2018). However, it is not clear whether SHBG might exert its effects on a transcriptional level only rather than posttranslational, as no significant changes in FASN or SCD1 protein levels were observed in the present study.

While adipocyte hypertrophy has been clearly pointed as a pivotal contributor of obesity and metabolic syndrome associated adipose tissue dysfunction and systemic metabolic failure, hyperplasia derived from adipocytes precursors differentiation has been proposed as a potential anti-obesity protective mechanism, where increased number of adipocytes is believed to attenuate the lipids engorgement (Ghaben and Scherer, 2019). However, several lines of evidence also indicated that excessive adipogenesis may trigger immoderate fat accumulation and contribute to adipose tissue inflexibility (Jakab et al., 2021). In this investigation, EMS SAT displayed disrupted adipogenic regulatory network, where depleted expression of adipogenesis repressors has been observed in favour of pro-adipogenic initiators profusion. In the past few decades, several transcription factors have been identified as either stimulators or repressors for adipogenesis in white adipose tissue. Although C/EBP family members and peroxisome proliferator-activated receptor γ (PPARγ) are known as master adipogenesis mediators expressed at various differentiation stages, other important pathways are also involved in the initiation of adipogenic cascades. Among others, the Kruppel-like factors KLF4, -9, and -15 positively regulate the expression of pro-adipogenic genes including PPARγ and C/EBPα/β/δ (Ahmad et al., 2020). Likewise, the STAT5A protein has been implicated in the regulation of various cellular processes including adipogenic differentiation, by conveying the extracellular pro-adipogenic stimuli to the nucleus to initiate PPARγ transcription (Wakao et al., 2011). Conversely, other signaling molecules have been appointed as direct adipogenesis suppressors. KLF2, -3, -7 and -16, as well as β -catenin (CTNNB1) have thus been reported to specifically alter the expression of C/EBPα, PPARγ and sterol regulatory binding protein-1c (SREBP-1c) and inhibit the percussor cells commitment into mature adipogenic lineage (Pollak et al., 2018; Chen et al., 2020). Here we found that incubation of EMS SAT with exogenous SHBG promoted the expression of adipogenesis repressors, namely, KLF3, IRF3 and CTNNB1, while downregulating the regulators of PPARγ and C/EBPα that include KLF4, KLF9, STAT5A and CEBPD. These data suggest a potential antiadipogenic property of SHBG, which has been similarly reported by Yamazaki et al. (2018), in their study, where they showed that SHBG suppressed the expression of CEBPα, PPARγ, and SREBP1 as key transcription factors controlling adipogenesis in 3T3-L1 preadipocytes. In another study, lower SHBG levels have been strongly correlated with increased adipogenesis, however in liver tissue of patients with obesity and metabolic syndrome (Xing et al., 2022), providing a strong evidence for the potential regulatory effect of SHBG towards adipogenesis. To our best knowledge, this is the first study showing the influence of SHBG on the expression profile of pro- and anti-adipogenic mediators in EMS white adipose tissue, and further investigations are needed to fully elucidate the exact molecular targets underlying the anti-adipogenic effects of SHBG.

Insulin signalling impairment as a core mechanism underlying EMS onset negatively impacts the lipid metabolism within adipocytes. A critical defect in post-insulin receptor’ signalling events including tyrosine phosphorylation of IRS proteins and activation of Pi3K-Akt axis has been further reported in adipocytes derived from obese subjects (Czech et al., 2013). In this study, the disrupted lipid metabolism has been found accompanied with significant blunted insulin signalling in EMS SAT. Proteins profiling revealed a substantial decrease in both INSR, IRS1/2, Akt and Pi3K phosphorylation levels, indicating a strong insulin desensitization. Impaired lipolysis and resulting hypertrophy of adipocytes has been associated with systemic insulin resistance and elevated circulating insulin levels under metabolic syndrome condition (Arner et al., 2010). In subcutaneous adipose tissue, insulin is an important adipogenic hormone mediating the FFAs storage via triglycerides biosynthesis within the adipocytes (Gastaldelli et al., 2017). Thus, previous data demonstrated that IR and associated improper IRS/Pi3K/Akt transducers phosphorylation significantly hampers the activity of main lipogenic enzymes including LPL and FASN, and loss in insulin-mediated cascades trigger to profound defective triglyceride clearance and overall dysregulated fatty acids and triacylglycerol metabolism (Panarotto et al., 2002; Mayas et al., 2010; Wong and Sul, 2010). Interestingly, the observed ability of SHBG to regulate the levels of LPL and FASN has been with a visible amelioration of the IR, Pi3K, IRS1/2 and Akt phosphorylation status of EMS SAT. Furthermore, we found that application of SHBG to EMS SAT increased the protein level of Glut-4, suggesting a possible alleviation of insulin resistance and improvement of the glucose uptake capacity. Similarly to our observations, decreased SHBG mRNA and protein levels have been previously correlated to lower expression of insulin-associated effectors, namely, IRS-1, IRS-2, PI3Kp85α and GLUT-3 and GLUT-4, and has been further linked to the PI3K/AKT pathway-mediated systemic insulin resistance (Feng et al., 2018). Moreover, SHBG overexpression has been reported to enhance the Glut-1-mediated glucose uptake and utilization via cAMP/PKA/CREB1 pathways activation in a human model of insulin resistant placental trophoblasts (Chi et al., 2020). What is more, Robker and others (Robker et al., 2009), that obesity phenotype of granulosa and cumulus cells correlated with increased insulin levels and suppressed SHBG expression in combination to lower Glut-4 and IRS2 transcript abundance, evoking an IRS1/IRS2 axis deactivation and a resulting insulin resistance status. Remarkably, the same study evidenced an additional connection between depleted SHBG levels and loss in ChREBP and SREBP-1, two critical mediators involved in lipogenesis regulation and lipogenic enzymes modulation, substantiating the prospective ability of SHBG to enhance insulin and lipid homeostasis in EMS SAT.

Increased adiposity and hyperinsulinemia has been closely linked to low-grade systemic inflammation in the course of EMS, resulting from the excessive production and release of various adipokines including the proinflammatory cytokines IL-1, IL-6, and the tumor necrosis factor (TNF) α (Vick et al., 2007). Here we found that EMS SAT was characterized by significant increased levels of IL-6 and moderate elevated PGE2 and MCP-1. Additionally, lipidomic analysis showed a critical elevation in polyunsaturated fatty acids (PUFAs) notably arachidonic and linoleic acids as well as eicosanoids, all known as important inflammation contributors. Indeed, under metabolic syndrome and hyperglycaemic condition, linoleic acid has been proposed to influence inflammatory reactions upon its Lipoxygenases (LOX)-mediated oxidation to highly reactive derivatives, namely, hydroxyoctadecadienoic acids (HODEs), oxo-HODEs and epoxy-HODEs (Vangaveti et al., 2016). Linoleic acid is further engaged in arachidonic acid biosynthesis via its conversion to γ-linolenic acid by the Δ^6^-desaturase (Norris and Carr, 2021). Under unfavourable conditions such as metabolic distress, arachidonic acid undergoes rapid conversion to endoperoxides that are used by cyclooxygenases to synthetise a pleiotropy of eicosanoids including prostaglandins, thromboxanes, leukotrienes, and lipoxins (Litwack, 2022). Arachidonic-derived eicosanoids have been reported as highly active pro-inflammatory mediators, and earlier investigations demonstrated that specific overactivation of cyclooxygenase 2 (COX2) triggers severe inflammatory response in the adipose tissue of obese subjects with high arachidonic acid levels. Likewise, upregulated COX2 has been correlated with an activation of the JNK/NFκB pathway leading to MCP-1 and IL-6 induction and immune cell infiltration into adipose tissue (Gartung et al., 2016). Hither, the *ex-vivo* treatment of EMS SAT with 50 nM SHBG exerted a potent anti-inflammatory effect as evidenced by the noticeable lowered levels of pro-inflammatory markers IL-6, TNF-α and MCP-1. The observed effect positively correlates with a number of reports that suggested the potential use of SHBG as an inflammation in the course of metabolic syndrome. Chronic inflammatory diseases such as polycystic ovary syndrome (PCOS), diabetes or obesity have been associated with low circulating SHBG levels in female patients (Ding et al., 2009). Withal, Yamazaki and collaborators (Yamazaki et al., 2018), in like manner found that SHBG efficiently abolished lipopolysaccharide- (LPS-) induced inflammatory response by suppressing the release of MCP-1, TNFα, and IL-6 cytokines in adipocytes and macrophages, along with phosphorylations of JNK and ERK. Interestingly, we also found that SHBG decreased the tissue levels of arachidonic and linoleic acids, which might have contributed to its observed anti-inflammatory effect, and further stands in line with the formerly underscored interrelation between high pro-inflammatory polyunsaturated fatty acids and low SHBG levels in adult human subjects (Liu et al., 2022).

Notably, our study evidenced the activation of both protein disulfide-isomerase A3 (PDI3A) stress and extracellular signal-regulated kinase 1/2 (ERK1/2) kinases in the SAT biopsies derived from EMS horses. Initially identified as a stress response molecule and a protein chaperone mediating the unfolded protein response (UPR), PDIA3 has been later showed to negatively impact various cellular processes in disease. Hence, accumulating evidence demonstrate that PDIA3 is promoting immune-activated hallmarks, and its depletion inhibits pro-inflammatory responses and oxidative damage (Wang et al., 2019). Besides, ERK1/2 plays an important role in determining cell survival or death, it has also been wildly described as an important regulator of pro-inflammatory gene expression including JNK/NF-κB signaling pathways, IL-1β, IL-6 and TNF-α cytokines (Li et al., 2016). Accordingly, SHBG treatment engendered a visible downregulation of both PDIA3 and ERK1/2 in the EMS SAT, which might represent a possible molecular mechanism underlying the anti-inflammatory activity of SHBG. In point of fact, SHBG has been thus far described as a direct inhibitor of ERK pathway markers activation (Wang et al., 2020). Moreover, previous data emphasized the stimulatory effect of PDIA3 toward ERK1/2 activation, as PDIA3 silencing results in a critical ERK depletion (Ye et al., 2018). Taken together, these findings infer that SHBG may exert its biological functions by regulating the ERK pathway components through PDIA3 modulation.

## 5 Conclusion

Equine metabolic syndrome strongly impairs the endocrine properties of subcutaneous adipose tissue by altering lipid metabolism homeostasis and promoting low-grade inflammation, which collectively trigger insulin resistance in relation to depleted SHBG levels. In this investigation, we evidenced the great potential of SHBG treatment in restoring proper SAT physiology by enhancing the expression of lipogenic enzymes and regulating the overall lipid profile. Moreover, SHBG application exerted a strong insulin sensitizing effect and showed remarkable anti-inflammatory potential through the modulation of the pro-inflammatory cytokines IL-6, TNF-α and MCP-1 and the reduction of arachidonic and linoleic acids levels. Interestingly, we demonstrated for the first time that SHBG may target the PDIA3/ERK axis to attenuate the exaggerated inflammatory responses and aberrant lipid metabolism inflexibility. This research therefore contributes to understanding the possible intercorrelation between SHBG protein and equine metabolic syndrome, and provides new molecular insight into the mechanism by which SHBG exerts its metabolic regulatory action.

## Data Availability

The original contributions presented in the study are included in the article/supplementary material, further inquiries can be directed to the corresponding author.
